# Individual participant data meta-analysis of eating behaviour traits as effect modifiers in acceptance and commitment therapy-based weight management interventions

**DOI:** 10.1038/s41366-025-01759-9

**Published:** 2025-04-10

**Authors:** Laura Kudlek, Patricia Eustachio Colombo, Julia Mueller, Stephen J. Sharp, Clare E. Boothby, Simon J. Griffin, Meghan Butryn, Christina Chwyl, Evan Forman, Charlotte Hagerman, Misty Hawkins, Adrienne Juarascio, Bärbel Knäuper, Marjukka Kolehmainen, Michael E. Levin, Jason Lillis, Edurne Maiz, Stephanie Manasse, Lara Palmeira, Kirsi H. Pietiläinen, Nancy E. Sherwood, Amy L. Ahern

**Affiliations:** 1 MRC Epidemiology Unit, University of Cambridge, Cambridge, UK; 2 Department of Psychology, Drexel University, Philadelphia, PA USA; 3 Department of Health and Wellness Design, School of Public Health, Indiana University, Bloomington, IN USA; 4 Department of Psychology, McGill University, Montreal, Quebec Canada; 5 Institute of Public Health and Clinical Nutrition, University of Eastern Finland, Kuopio, Finland; 6 Department of Psychology, Utah State University, 2810 Old Main Hill, Logan, UT 84322 USA; 7 The Department of Psychiatry and Human Behavior, Warren Alpert Medical School of Brown University, Providence, Rhode Island USA; 8 The Weight Control and Diabetes Research Center, The Miriam Hospital, Providence, Rhode Island USA; 9 Department of Clinical and Health Psychology and Research Methodology, University of the Basque Country UPV/EHU, Donostia-San Sebastian, Basque Country, Spain; 10 RISE-Health, Department of Psychology and Education, Universidade Portucalense Infante D. Henrique, Rua Dr. António Bernardino de Almeida, 541, 4200-072 Porto, Portugal; 11 Obesity Research Unit, Research Program for Clinical and Molecular Metabolism, Faculty of Medicine, University of Helsinki, Helsinki, Finland; 12 Healthy Weight Hub, Abdominal Center, Helsinki University Hospital, Helsinki, Finland; 13 Division of Epidemiology and Community Health, School of Public Health, University of Minnesota, Minneapolis, Minnesota USA

**Keywords:** Weight management, Nutrition therapy, Risk factors

## Abstract

**Background:**

Obesity care may benefit from precision approaches, matching patients to treatment types based on their individual characteristics, including eating behaviour traits (EBTs) like emotional eating, uncontrolled eating, external eating, internal disinhibition and restraint. Initial evidence suggests that Acceptance and Commitment Therapy (ACT)-based interventions might address dysregulated EBTs more effectively than standard behavioural treatments. However, it is unclear if ACT is more effective for certain EBT levels.

**Methods and analysis:**

This pre-registered (CRD42022359691) one-stage Individual Participant Data (IPD) meta-analysis explored the moderating effects of baseline EBTs on weight outcomes in trials of ACT-based interventions for adults with a BMI ≥ 25 kg/m^2^. Unlike traditional meta-analyses, IPD meta-analyses re-analyse existing data to answer novel research questions. We identified 16 eligible trials through a systematic search of eight databases until June 20, 2022. We obtained, checked, and harmonised data from 15 trials (*N* = 2535). We used mixed regression models to investigate both continuous and categorical interaction effects.

**Results:**

We found no evidence of interactions between ACT vs. control and baseline EBTs as continuous variables on percentage weight change. However, we found evidence to suggest an added difference in weight change of −4.47% (95%CI −1.15, −7.73) from baseline to 12-months after intervention end in participants with medium levels of internal disinhibition compared to those with high levels. Sensitivity analyses similarly indicated a greater intervention benefit for participants with medium, rather than high, emotional eating levels (in trials that reduced experiential avoidance and in trials using the three-factor eating questionnaire) and internal disinhibition (in analyses of participants with at least 60% attendance). Given the exploratory nature of analyses, results should be interpreted with caution.

**Conclusion:**

Findings suggest potential non-linear interaction effects of ACT with internal disinhibition but require replication in confirmatory trials. These results may help guide further research on precision approaches based on EBTs.

## Introduction

Obesity increases the risk of developing diabetes, cardiovascular diseases, certain cancers and other metabolic disorders, contributing to a reduced quality of life and higher mortality rates [1]. As the prevalence of obesity has drastically increased globally over the past few decades [1, 2], innovative treatment approaches are needed to improve health outcomes on a large scale and reduce costs to healthcare and society. Although weight loss can successfully be achieved through a variety of treatments, such as behavioural weight management interventions (BWMIs), treatment outcomes vary significantly among individuals [3]. Precision treatment approaches aim to allocate individuals to treatment types they are most likely to benefit from, which has the potential to improve both efficiency and effectiveness of treatment options [4, 5]. For example, we may allocate types of BWMIs to people seeking treatment based on characteristics that causally contribute to individual differences in treatment response [6–8].

Eating Behaviour Traits (EBTs) are thought to contribute to individual treatment variability [9–13] and have been linked to a higher BMI and weight gain [14–21]. They can be understood as psycho-behavioural patterns in response to food and food-related cues that determine the type, amount, and frequency of food consumed, as well as when to start and stop eating [22, 23]. In contrast to fluctuating states of hunger and fullness, EBTs reflect stable tendencies that are thought to predispose individuals to energy intake more systematically [24]. A range of partly overlapping EBTs are typically investigated in the context of weight management, and capture tendencies such as eating in response to emotional or external cues and experiencing a loss of control over food intake [25–27]. Baseline levels of these traits may indicate how well a certain type of intervention works for these participants. For example, people with high baseline levels of food responsiveness, also known as external eating and referring to the tendency to react strongly to the presence, sight, smell or taste of food, were found to benefit more from a custom BWMI that specifically aimed to target this EBT compared to an active standard BWMI control condition including dietary advice and common behaviour change techniques (e.g. self-monitoring, goal setting, meal planning, problem solving) [12].

Other types of BWMIs that are more commonly investigated in the context of targeting EBTs include Acceptance and Commitment Therapy (ACT)-based interventions. ACT aims to increase psychological flexibility, which describes the ability to remain in the present moment and experience thoughts and feelings without avoidance or judgement while taking actions that align with core values, in order to address a wide range of problem areas including mental health and health behaviour change [28]. BWMIs that include ACT components can support people to accept cravings and negative emotions and help them to understand what triggers their overeating [29]. This can reduce reliance on food to relieve urges and regulate emotions, thereby addressing a number of dysregulated EBTs, such as emotional eating (i.e. the tendency to overeat in response to negative emotions) and disinhibition (i.e. the tendency to experience a loss of control over eating) [30]. Forman et al. previously evaluated whether the effect of ACT-based BWMIs compared to an active standard BWMI control condition depended on participants’ EBT levels. They found such moderating effects for high versus low emotional eating and disinhibition in one study, [31] but not for disinhibition in another [32]. To our knowledge, no other trials have investigated the moderating effects of EBTs in ACT-based interventions.

The scarcity of research in this area, which may in-part be attributable to a lack of trials powered to investigate effect modifiers, makes it challenging to derive practical recommendations for precision treatment approaches. Individual Participant Data (IPD) meta-analysis can address issues of power by collecting and re-analysing raw data from relevant trials. In contrast to traditional aggregate meta-analyses that summarise existing findings, IPD meta-analyses create a resource to investigate novel research questions, much like secondary analyses of existing datasets. Thus, in this study, we aimed to investigate whether the effect of ACT-based interventions on weight loss is moderated by participants’ baseline levels of EBTs in an IPD meta-analysis. Findings may contribute to a new understanding of the potential use of ACT-based interventions in a precision treatment approach based on EBT levels.

## Methods

We followed guidance from the Preferred Reporting Items for Systematic Reviews and Meta-Analyses for IPD extension (PRISMA-IPD) (Supplementary Material Table SM 1-1) [33]. Ethical approval was obtained from the Cambridge Psychology Research Ethics Committee (Application No: PRE.2023.121). We pre-registered the study on PROSPERO (CRD42022359691) and a study protocol was published in BMJ Open [34]. Additionally, we uploaded a pre-specified analysis plan to the Open Science Forum (OSF) (10.17605/OSF.IO/3X6YM). Due to small sample sizes and lack of covariate variation within some studies, we switched from the initially planned two-stage approach to a one-stage approach. This provided a larger combined sample size and allowed for a more practical approach when adjusting models by automating the inclusion of available covariates in individual studies.

### Study identification and selection

Eligible studies were both published or unpublished studies on adults (>18 years) with overweight or obesity attending an ACT-based intervention with the primary goal of weight loss or weight loss maintenance that included bodyweight and EBT data. Eligible EBTs were:

- *Emotional Eating* (i.e. the tendency to overeat in response to negative emotions).
- *External Eating* (i.e. the tendency to overeat in response to environmental cues, such as the sight, smell or taste of food).
- *Disinhibition* (*general disinhibition* [i.e. a general tendency for loss of control eating in response to a variety of emotional, cognitive and environmental cues], *internal disinhibition* [i.e. loss of control eating in response to cognitive and emotional cues], *external disinhibition* [i.e. loss of control eating in response to environmental cues, such as sight, smell or taste]).
- *Restraint* (*general restraint* [i.e. the conscious control of food intake with the aim to influence body weight], *flexible restraint* [i.e. adaptive control of food intake allowing for eating all foods in moderation within a structured eating plan], *rigid restraint* [i.e. strict control of food intake excluding foods or food groups to influence body weight]).
- *Uncontrolled Eating* (i.e. the tendency to overeat impulsively in response to a variety of internal and external cues and experience loss of control over food intake).

These traits were selected given their frequent assessment by well-established questionnaires such as the Three Factor Eating Questionnaire (TFEQ) and Dutch Eating Behaviour Questionnaire (DEBQ). Disordered eating was excluded to retain focus on non-clinical manifestations of EBTs, although we acknowledge that EBTs like emotional and uncontrolled eating may lie on the lower levels of the same spectrum as binge eating disorder [35].

Any study type was included for the overall IPD project; however, only randomised controlled trials (RCTs) were eligible for inclusion in the current paper exploring the moderating role of EBTs in ACT-based BWMIs. A detailed description of PICOs is provided in Table SM 2-1.

The search strategy was based on a review by Lawlor et al. [36] on third wave cognitive behavioural therapies for weight management. We re-ran an adaption of their search (that removed concepts relating to therapies other than ACT) in 8 databases (MEDLINE, CINAHL, Embase, PsycINFO, AMED, ASSIA, Web of Science, CENTRAL) until 20th June 2022 and conducted hand-searches of reference lists of key publications. The full search strategy is provided in the Supplementary Material Section 3.0.

Two independent reviewers, PEC and LK, screened both studies included in Lawlor et al. and newly identified records against this project’s eligibility criteria in Covidence at both title and abstracts and full text screening stage [37]. We resolved disagreements by consultation of a third reviewer, AA, where necessary. We contacted authors of studies where eligibility was unclear or where studies were ongoing to enquire about authors willingness to share unpublished data.

### Data collection and management

#### Requesting and collecting IPD

We contacted authors via email to invite them for collaboration and data sharing. After initial contact, we sent two reminders, each with a time-period of around 3 weeks between them. If no response was provided or contact was lost after two reminders, we excluded the study.

We set up data sharing agreements with institutions of collaborating authors and shared a detailed data dictionary of requested variables prior to data transfer (ID, age, sex/gender, height, trial arm, number of sessions attended, exclusion [if applicable] and reason for exclusion, weight, EBTs, and experiential avoidance at baseline, end of intervention, and any available follow-up). Data sharing strictly followed the conditions pre-specified in data transfer agreements and we stored data in a locked folder on the MRC Epidemiology Unit’s research drive.

Any study-level data required for analyses or description of study characteristics were extracted from published manuscripts by two reviewers (LK, PEC) independently, using an adaption from the Cochrane data extraction form (Supplementary Material Section 4.0) [38]. This included data on study design and setting, participant sociodemographic characteristics (e.g. ethnicity, education, socioeconomic status) and details on the intervention and control conditions. A third reviewer (JM) cross-checked extractions for any discrepancies, and we provided original study authors with an opportunity to cross-check extractions for accuracy, including any potential updates of ongoing studies.

#### Data harmonisation and checking

We harmonised data according to the pre-specified data dictionary shared with collaborators. This involved re-coding and transforming variables under a uniform coding scheme (e.g. we transformed height and weight into metric units, age into age in years, and EBTs into a common scale ranging from 0–100, where 0 represents the lowest possible score and 100 the highest possible score). We considered EBTs assessed by different questionnaires as conceptually equivalent and treated them as one trait if considerable overlap between items was present (e.g. emotional eating from the DEBQ and TFEQ). Where assessment differed considerably, these were considered separate traits (e.g. emotional eating from the TFEQ and the Emotional Eating Scale [EES]—Anger and Frustration subscale).

Data checking was conducted on three levels. Firstly, wherever item-level data was available for questionnaires, we recomputed subscale scores and compared them to author-computed ones to confirm item scoring and ensure the uniform allocation of items to subscales. Secondly, we compared descriptives derived from IPD to those reported in the original publication. Thirdly, we created a merged dataset containing harmonised data from all included studies for analyses and checked descriptives pre- and post-merging to ensure that no errors were introduced in the merging phase.

Any discrepancies were discussed with study authors. If discrepancies could not be resolved, we considered the severity of the deviation. In case of low to moderate deviations, we flagged the trial in question in the risk of bias assessment and excluded it during sensitivity analyses (see section “Risk of bias”).

Data cleaning, harmonising and merging was done in Stata v17 [39].

#### Studies where IPD is not available

If authors of eligible studies did not provide IPD, we excluded the respective study. Reasons for non-provision are summarised in Table SM 5-1. We extracted published data on sociodemographic data, EBTs and weight loss outcomes in duplicate using a pre-specified data extraction form and summarised study characteristics in Table SM 7-2 for comparison to included studies.

### Risk of bias assessment

We used the Cochrane Risk of Bias tool 2 (RoB2) [40] to assess risk of bias within studies. The tool was used by two researchers independently (LK, and either PEC or JM). Disagreements were resolved by discussion and consulting of a third reviewer (AA) if necessary. Missing information (e.g. for ongoing studies) was sought from authors directly. Since we re-analysed IPD as opposed to relying on reported outcomes, domain 5 (selection of reported results) of the RoB2 tool was not considered applicable and removed from the overall assessment. Additionally, we added a domain describing data discrepancies occurring during data checking (see section “Data harmonisation and checking”). If data discrepancies were only observed for some, but not all EBTs, the RoB was classified differently depending on the moderator of interest.

Risk of bias may also arise from the accumulated body of evidence, such as not obtaining IPD of particular studies. We evaluated this by comparing study characteristics, outcomes, and risk of bias assessments within studies of trials not providing IPD to those who did (see section “Studies where IPD is not available”).

### Data analyses

#### Descriptive statistics

Descriptive statistics were derived from the raw data directly and reported separately for intervention and control groups of each study. Other study and intervention characteristics, for which IPD was not requested, were extracted from published reports.

#### Statistical analysis

Analyses followed guidance on IPD meta-analysis by Riley et al. [41]. We explored effects of interest using a one-stage IPD meta-analysis. In contrast to a two-stage approach, where analyses of interest are first conducted in each included trial separately and then synthesised using traditional meta-analyses, a one-stage approach is conducted on the pooled sample while controlling for clustering within studies. We used mixed effects regression models for all analyses estimating effects of interest using a restricted maximum likelihood approach (REML). We first estimated the overall effect of the intervention (ACT vs. control) on (1) percentage weight change and (2) on changes in EBTs by specifying a random intervention effect and stratifying all other parameters by study, including intercepts and individual-level covariates (age, sex, baseline weight/baseline EBT). A further study-level covariate, intervention duration, was added as a common effect, as it could only vary between and not within studies. Stratifying nuisance parameters (i.e. all parameters other than the effect of interest) ensured that clustering within the different studies was accounted for, closely resembling a two-stage approach.

We used a similar model structure to investigate the moderating effects of EBTs, where we included the interaction between randomised group (ACT vs. control) and EBTs as a random effect, while stratifying nuisance parameters by study. This ensured that the interaction reflected within-trial effects only and removed the need to include a between-trial interaction term [41]. We adjusted for age, sex, baseline weight, baseline EBT values, and intervention duration in the interaction models. To explore potential non-linear interactions of EBTs and randomised group, we also fit another mixed effects regression model with EBT as a categorical variable (reflecting *“low”, “medium”*, and *“high”* levels of the respective EBT, using medium as the reference category). Categorisation was based on using the tertile EBT scores of the pooled sample (excluding those trials where discrepancies were observed during data checking) as cut-offs (Table SM 11-1). Finally, we estimated the effect of the intervention on percentage weight change in strata of participants with *“low”, “medium”*, and *“high”* levels of each EBT to facilitate the interpretation of potential interaction effects.

All effects were estimated on percentage weight change (1) from baseline to the end of intervention, (2) from baseline to 6 months after the end of intervention, and (3) from baseline to 12 months after the end of intervention. Analyses were based on a complete-case approach, and were only conducted for EBTs with five or more contributing studies. Confidence intervals were calculated using likelihood profiling. Given the exploratory nature of analyses, we did not consider *p* values for interpretation of significance and rather used confidence intervals to report effects of interest. We did not adjust for multiple testing, but instead focused our interpretation of results on the strength, direction and consistency of effects. This approach has been recommended for exploratory analyses like this as compared to studies with a confirmatory objective [42]. Heterogeneity is represented by the between-study variance (tau^2^) of the random interaction effect.

Analyses were performed in R 4.1.3 [43] using the ‘lmer’ function from the ‘lme4’ package [44] and the ‘lmerTest’ package [45].

#### Sensitivity analyses

We conducted a series of sensitivity analyses for the investigation of moderating effects of EBTs. At the study-level, we compared results from analyses performed in the full set of trials versus (1) trials classified as low risk of bias, (2) trials with waitlist or minimal control conditions, (3) trials with an active standard BWMI control condition and (4) trials that significantly reduced experiential avoidance. At the individual level, we compared results from analyses performed in all individuals versus (5) those that received a sufficient dose of the intervention, considered to be equal to an attendance of at least 60% of sessions. Trials that did not measure variables of interest were not considered for sensitivity analyses. Sensitivity analyses were only conducted if more than 500 observations were available for interaction models.

### Patient and Public Involvement and Engagement (PPIE)

PPIE input was sought for the reflection on results and identification of implications for practice. We arranged remote meetings with two PPIE members reporting lived experience of obesity and dysfunctional eating behaviour. Meetings were of one hour duration each and included a brief presentation of findings and an in-depth brainstorming exercise facilitated by probing questions. Insights generated in those meetings were incorporated into the discussion.

## Results

### Study selection and characteristics

**Fig. 1:**
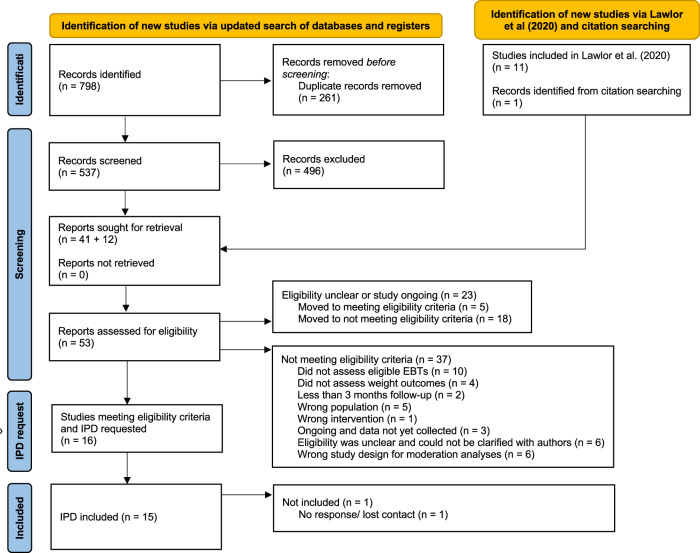
Flowchart depicting the study selection process.

IPD was obtained from a total of 15 out of 16 trials eligible for the present study [31, 32, 46–58] amounting to 2535 observations in total. IPD could not be obtained from one trial due to a loss of contact (Table SM 5-1) [59]. A flowchart of the study selection process is depicted in Fig. 1. An overview of study characteristics is displayed in Table 1 and detailed study characteristics are described in the Supplementary Materials Table SM 7-1. Participant characteristics and baseline EBT levels are summarised in Supplementary Tables SM 9-1 and 10-1, respectively. Additionally, the observed EBT scores are plotted against percent weight change in Figs. SM 12-1–SM 12-6.

**Table 1:** Overview of characteristics of included trials.

Study characteristics	*N* Studies	Citations
Trial status		
Completed	10	[31, 32, 49–54, 56, 57]
Ongoing	5	[45–48, 55]
Overall risk of bias rating		
Low	8^a^	[31, 32, 46, 49, 50, 52, 54, 57]
Some concerns	7	[45, 47, 48, 51, 53, 55, 56]
High	0	n/a
Study location		
United States of America	9	[31, 32, 46–49, 51, 54, 57]
United Kingdom	2	[55, 56]
Finland	1	[52]
Portugal	1	[50]
Spain	1	[45]
Canada	1	[53]
Comparison type^b^		
Gold Standard (behavioural)	8	[31, 32, 46–49, 54, 57]
Usual care	5	[45, 50, 53, 55, 56]
Waitlist	4	[50–52, 56]
Comparison intensity		
Low	4	[51, 52, 55, 56]
High	8	[31, 32, 46–49, 54, 57]
Unknown^c^	3	[45, 50, 53]
Intervention duration		
≤12 weeks	3	[51, 52, 56]
12–26 weeks	4	[45, 50, 53, 55]
≥26 weeks	8	[31, 32, 46–49, 54, 57]
Intervention delivery mode		
Face to Face	8	[31, 32, 45, 46, 50, 53, 54, 57]
Remote	4	[47, 51, 55, 56]
Mixed	3	[48, 49, 52]
Intervention delivery format		
Individual	4	[51, 53, 55, 56]
Group	7	[31, 45–47, 50, 54, 57]
Mixed	4	[32, 48, 49, 52]
EBT Questionnaire used^d^		
TFEQ - 51	7	[31, 32, 47–49, 54, 57]
TFEQ - R18 or TFEQ - R21	8	[32, 47, 50–52, 54–56]
DEBQ	3	[31, 45, 53]
EOQ	1	[49]
EES	2	[31, 46]

*N* number of, *n/a* not applicable, *EBT* eating behaviour trait, *TFEQ* Three Factor Eating Questionnaire, *DEBQ* Dutch Eating Behaviour Questionnaire, *EOQ* Emotional Overeating Questionnaire, *EES* Emotional Eating Scale.

^a^Butryn 2022 was classified as some concerns only for the emotional eating outcome due to data discrepancies.

^b^Multiple categories possible (e.g. usual care and waitlist).

^c^Comparison intensity may be unknown for participants in usual care groups where healthcare support is provided and may depend on individuals’ insurance, etc.

^d^Questionnaires might not have been administered in full, but only for select EBT subscales.

The majority of included trials was conducted in the US (*n* = 9) [31, 32, 47–50, 52, 55, 58], followed by other high-income countries such as the UK (*n* = 2) [56, 57], Canada (*n* = 1) [54], Finland (*n* = 1) [53], Portugal (*n* = 1) [51], and Spain (*n* = 1) [46]. Sample sizes of these trials ranged from 58 [56] to 369 [57]. Most participants were female (81%) and ages ranged from 18 to 84 years with a mean (SD) age of 50 (11.57). The mean BMI was 35 kg/m^2^ (5.72), ranging from 24 to 65 kg/m^2^.

ACT-based interventions had a mean duration of 37 (23.21) weeks, with the shortest being 8 weeks and the longest 78 weeks. The majority of interventions were conducted in face-to-face (*n* = 8) [31, 32, 46, 47, 51, 54, 55, 58] and group settings (*n* = 7) [31, 46–48, 51, 55, 58]. More recent trials tended to be remote (*n* = 4) [48, 52, 56, 57] and targeted towards the individual (*n* = 4) [52, 54, 56, 57].

Out of the eligible EBTs and their subscales, data were available from five or more contributing studies for:

- Emotional eating, as assessed by the TFEQ-21 Item Version [51, 56, 57], the TFEQ-18 Item Version [32, 48, 53, 55], the Emotional overeating Questionnaire (EOQ) [60], the DEBQ [31, 46, 54], and the EES-Anxiety and Depression subscale [47].
- External eating/disinhibition, as assessed by the DEBQ [46, 54] and TFEQ-51 Item Version [31, 32, 48–50, 55, 58].
- Internal disinhibition, as assessed by the TFEQ-51 Item Version [31, 32, 48–50, 55, 58].
- Restraint, as assessed by the TFEQ-21 Item Version [51, 56, 57], the TFEQ-18 Item Version [32, 48, 52, 53, 55], the TFEQ-51 Item Version [31, 49, 58] and the DEBQ [46, 54].
- Uncontrolled eating, as assessed by the TFEQ-21 Item Version [51, 56, 57] and the TFEQ-18 Item Version [32, 48, 52, 53, 55].

External eating, assessed by the DEBQ, and external disinhibition, assessed by the TFEQ-51, were treated as the same trait due to the conceptual overlap and similarity of questionnaire items. Although there are conceptual similarities between other traits, such as emotional eating, internal disinhibition and uncontrolled eating, we did not consider these sufficient to warrant a merging of traits and instead examined them separately to capture potential nuanced differences.

### IPD integrity

No major data discrepancies were observed during data checking. However, we were unable to compare all IPD against published data, as relevant variables were sometimes presented in non-replicable formats (e.g. using imputed data or reflecting different sub-samples) and EBTs were often not reported as outcome of interest in the included trials. Similarly, no publications were available for data checking in ongoing trials. One trial had noticeably low average emotional eating scores, potentially due to the use of a different questionnaire [50]. This discrepancy was reflected in risk of bias assessment for the emotional eating outcome, and we removed the respective trial from relevant sensitivity analyses (Fig. SM 6-1).

Observations that were excluded by original trial authors were also treated as excluded for the present IPD analyses. Additionally, we removed biologically implausible values, as pre-defined in the analysis plan (10.17605/OSF.IO/3X6YM). Table SM 8-1 shows how many observations were excluded in each trial and for which reason.

### Risk of bias within and across studies

Eight trials were classified as having low risk of bias, with the remaining seven being considered to have some concerns (Fig. SM 6-1). The most common reason for classifying a study as "*some concerns"* was the self-reported assessment of weight outcomes (*n* = 4), followed by baseline imbalances in bodyweight or EBTs (*n* = 2), missing outcome data (*n* = 1) and slight discrepancies identified during data checking (*n* = 1 for emotional eating).

The risk of bias across studies is judged to be low, as IPD could not be obtained for only one eligible trial. This trial was conducted in US veterans and although they found that the active standard BWMI control condition was more effective than the ACT condition at reducing weight, their sample size was low (*n* = 88) and likely underpowered [59]. The risk of bias across studies due to publication bias was further reduced as we included five ongoing and unpublished trials. However, it is important to note that five out of the 15 included studies were conducted at the same institution, potentially increasing the similarity among these studies compared to others [31, 32, 48, 50, 55].

### Intervention effect on weight change

There was a difference of mean percentage weight change from baseline to the end of intervention of −0.71% [95% CI −1.29, −0.03] in the ACT groups compared to control. At 6 months following the end of intervention, there was no evidence of an intervention effect on weight change (−0.28% [95% CI −1.32, 0.74]). At 12 months following the end of intervention, the largest significant difference of mean percentage weight change between intervention groups of −1.70% [95% CI −2.87, −0.53] was observed (Table SM 13-1).

### Intervention effect on changes in eating behaviour traits

We found evidence to suggest intervention effects on changes in emotional eating (−3.62 [95% CI −5.04, −1.71]), internal disinhibition (−3.24 [95% CI −5.93, −0.57]), restraint (2.86 [95% CI 1.08, 4.71]) and uncontrolled eating (−5.75 [95% CI −9.25, −2.51]) in the desired direction from baseline to intervention end. We found no evidence of an intervention effect on changes in external eating, although most values within the confidence interval were in the desired direction (−2.41 [95% CI −4.95, 0.12]) (Table SM 14-1).

### The moderating effects of EBTs on weight change

Tables 2–4 show estimates of the interaction of EBTs and randomised group on percentage weight change from baseline to intervention end (Table 2), from baseline to 6 months after intervention end (Table 3) and from baseline to 12 months after intervention end (Table 4), as well as intervention effects on percentage weight change in strata of low, medium, and high levels of EBTs. An overview of results from sensitivity analyses at end of intervention is provided in Tables SM 15-1–SM 15-5. The number of included studies and observations was too low to be further stratified for sensitivity analyses in the 6- and 12-months follow-up.

**Table 2:** Moderating effects of randomised group and eating behaviour traits on percent weight change at end of intervention.

Exposure	*N* sample [*N* studies]	Intervention effect on percentage weight change		Interaction of categorised EBTs and randomised group on percentage weight change		Interaction of continuous EBTs and randomised group on percentage weight change	
		MD (95% CI)^a^	Tau^2^	MD (95% CI)^b, c^	Tau^2^	MD (95% CI)^b^	Tau^2^
Emotional eating	1893 [13]					0.007 (−0.02, 0.04)	0.00
Low	809	−0.605 (−1.72, 0.53)	0.05	0.309 (−1.38, 2.06)	0.00		
Medium	565	−1.694 (−2.92, −0.65)	1.08	ref			
High	519	0.055 (−0.86, 1.48)	1.84	1.191 (−0.38, 3.21)	1.30		
External eating	1309 [9]					0.041 (−0.004, 0.09)	0.00
Low	578	−1.271 (−2.81, 0.26)	0.00	−0.418 (−2.71, 1.79)	1.42		
Medium	379	−0.899 (−2.72, 0.69)	1.88	ref			
High	352	1.213 (−0.4, 3.05)	1.21	1.577 (−0.91, 4.2)	0.00		
Internal disinhibition	1152 [7]					0.008 (−0.04, 0.06)	0.00
Low	413	−0.777 (−2.56, 1.13)	0.72	0.648 (−2.07, 3.21)	1.94		
Medium	397	−1.459 (−3.09, 0.41)	4.42	ref			
High	342	0.506 (−1.23, 2.46)	3.10	1.802 (−1.04, 4.46)	1.31		
Restraint	1786 [13]					−0.008 (−0.04, 0.03)	0.00
Low	745	−0.891 (−1.9, 0.12)	0.00	0.456 (−1.11, 2.03)	0.00		
Medium	472	−1.398 (−2.68, −0.11)	0.00	ref			
High	569	−0.932 (−1.81, 0.23)	0.92	0.373 (−1.27, 2.02)	0.00		
Uncontrolled eating	1328 [8]					0.004 (−0.03, 0.04)	0.00
Low	522	−1.423 (−2.63, −0.22)	0.00	−0.587 (−2.36, 1.19)	0.00		
Medium	386	−0.55 (−1.94, 0.84)	0.00	ref			
High	420	−1.538 (−2.57, 0.06)	1.85	−0.405 (−2.24, 1.49)	0.32		

Rank deficient coefficients were removed from the model by the lmer function (e.g. if one study only included females and no males).

*MD* mean difference, *CI* confidence interval.

^a^Intervention effects (ACT vs. control) in strata of low, medium, and high levels of EBTs on percentage weight change were estimated while stratifying all other parameters by trial.

^b^Interactions between randomised group (ACT vs. control) and EBTs on percentage weight change were estimated while stratifying all other parameters by trial.

^c^Reference group was medium levels of each EBT, respectively.

**Table 3:** Moderating effects of randomised group and eating behaviour traits on percent weight change at 6 months after the end of intervention.

Exposure	*N* sample [*N* studies]	Intervention effect on percentage weight change		Interaction of categorised EBTs and randomised group on percentage weight change		Interaction of continuous EBTs and randomised group on percentage weight change	
		MD (95% CI)^a^	Tau^2^	MD (95% CI)^b, c^	Tau^2^	MD (95% CI)^b^	Tau^2^
Emotional eating	722 [5]					−0.02 (−0.07, 0.03)	0.00
Low	448	0.022 (−1.62, 1.58)	0.00	0.318 (−3.03, 3.66)	0.00		
Medium	147	−1.117 (−3.81, 1.56)	0.75	ref			
High	127	−1.946 (−4.78, 0.94)	0.00	−1.254 (−5.18, 2.68)	0.00		
External eating	842 [6]					0.021 (−0.03, 0.07)	0.00
Low	337	−0.311 (−2.05, 1.43)	0.00	−0.572 (−3.27, 2.13)	0.00		
Medium	231	0.014 (−2.13, 2.15)	0.00	ref			
High	274	0.4 (−1.46, 2.26)	0.05	−0.123 (−3.03, 2.78)	0.00		
Internal disinhibition	719 [5]					−0.005 (−0.06, 0.05)	0.00
Low	236	0.082 (−2.02, 2.16)	0.00	0.214 (−2.99, 3.42)	0.00		
Medium	221	−0.039 (−2.45, 2.35)	0.00	ref			
High	262	0.133 (−1.79, 2.06)	0.05	0.537 (−2.65, 3.73)	0.00		
Restraint	614 [5]					−0.013 (−0.08, 0.05)	0.00
Low	253	−1.141 (−2.82, 0.98)	2.22	−0.068 (−2.81, 3.12)	3.29		
Medium	185	−1.232 (−3.49, 1.03)	0.00	ref			
High	176	−0.064 (−2.31, 2.19)	0.00	0.326 (−2.93, 3.52)	0.00		

Rank deficient coefficients were removed from the model by the lmer function (e.g. if one study only included females and no males).

*N* number of, *MD* mean difference, *CI* confidence interval.

^a^Intervention effects (ACT vs. control) in strata of low, medium, and high levels of EBTs on percentage weight change were estimated while stratifying all other parameters by trial.

^b^Interactions between randomised group (ACT vs. control) and EBTs on percentage weight change were estimated while stratifying all other parameters by trial.

^c^Reference group was medium levels of each EBT, respectively.

**Table 4:** Moderating effects of randomised group and eating behaviour traits on percent weight change at 12 months after the end of intervention.

Exposure	*N* sample [*N* studies]	Intervention effect on percentage weight change		Interaction of categorised EBTs and randomised group on percentage weight change		Interaction of continuous EBTs and randomised group on percentage weight change	
		MD (95% CI)^a^	Tau^2^	MD (95% CI)^b, c^	Tau^2^	MD (95% CI)^b^	Tau^2^
External eating	774 [6]					0.061 (−0.004, 0.13)	0.00
Low	335	−2.691 (−4.62, −0.75)	0.00	−1.055 (−3.81, 1.69)	0.00		
Medium	239	−1.978 (−4.15, 0.21)	0.00	ref			
High	200	−0.371 (−2.39, 1.62)	0.11	1.679 (−1.54, 4.91)	0.00		
Internal disinhibition	674 [5]					0.011 (−0.06, 0.08)	0.00
Low	193	−1.528 (−4.07, 1.02)	0.00	1.328 (−2.01, 4.64)	0.00		
Medium	262	−3.712 (−5.56, −1.41)	2.86	ref			
High	219	0.315 (−2.34, 1.78)	6.75	4.475 (1.15, 7.73)	8.50		
Restraint	776 [6]					−0.015 (−0.08, 0.05)	0.00
Low	330	−1.761 (−3.5, 0.09)	0.49	0.15 (−2.74, 3.04)	0.00		
Medium	214	−2.224 (−4.52, 0.07)	0.00	ref			
High	232	−1.714 (−3.8, 0.37)	0.00	−0.119 (−3.22, 2.99)	0.00		

Rank deficient coefficients were removed from the model by the lmer function (e.g. if one study only included females and no males).

*N* number of, *CI* confidence interval.

^a^Intervention effects (ACT vs. control) in strata of low, medium, and high levels of EBTs on percentage weight change were estimated while stratifying all other parameters by trial.

^b^Interactions between randomised group (ACT vs. control) and EBTs on percentage weight change were estimated while stratifying all other parameters by trial.

^c^Reference group was medium levels of each EBT, respectively.

We found no evidence of moderating effects when investigating the interaction between randomised group and EBTs as continuous variables on percentage weight change at any time point. However, it may be worth noting that for external eating, the confidence intervals of interaction estimates at end of intervention (0.04% [95% CI −0.004, 0.09]) and at the 12-month follow-up (0.06%, [95% CI −0.004, 0.13]) narrowly included zero, with most values suggesting a greater intervention benefit for lower levels of external eating. A similar direction and size of estimates was observed across sensitivity analyses for external eating, with the largest interaction estimate observed in sensitivity analysis of participants with at least 60% attendance (0.05% [95% CI −0.002, 0.10]) (Table SM 15-2). No consistent patterns were observed in sensitivity analyses for the interactions between the intervention and other continuous EBTs, including emotional eating, internal disinhibition, restraint, and uncontrolled eating.

In analyses investigating interactions of randomised group with EBTs categorised into *“low”, “medium”* and *“high”* levels, we found no evidence to suggest an effect on percent weight change at the end of intervention or at 6 months after intervention end. At 12 months after the end of intervention, we found evidence to suggest an interaction of randomised group with *"medium"* vs. "*high"* levels of internal disinhibition on percent weight change. Specifically, we found that those with medium levels of internal disinhibition benefited more from the intervention by displaying an additional 4.47% (95% CI 1.15, 7.73) decrease in percent body weight compared to those with high internal disinhibition levels (Table 4). Sensitivity analyses at intervention end resulted in similar patterns of estimates, indicating a greater benefit of ACT among participants with medium levels of internal disinhibition. The largest effect size was observed in the sensitivity analysis of participants with at least 60% attendance (3.75% [95% CI 0.39, 6.7]) (Table SM 15-3).

We found no evidence of moderating effects for the remaining categorised EBTs (emotional eating, external eating, restraint and uncontrolled eating) at any time point in the main analyses. However, we did find evidence of moderating effects in a number of sensitivity analyses for emotional eating at intervention end (i.e. in analyses of trials reducing experiential avoidance and in analyses of trials using the Three Factor Eating Questionnaire [TFEQ] to assess emotional eating) (Table SM 15-1). As with internal disinhibition, the direction and size of interaction estimates consistently suggested a greater benefit of ACT for participants with medium as compared to high levels of emotional eating. The lowest effect size with confidence intervals crossing zero was observed in trials comparing ACT to minimal control groups (0.33% [95% CI −0.94, 3.08]), and the largest effect size was observed in trials using the TFEQ (2.06% [95% CI 0.44, 4.01]) (Table SM 15-1).

## Discussion

This IPD meta-analysis combined data from 15 randomised controlled trials to investigate the moderating role of EBTs in ACT-based BWMIs. We found small but significant effects of ACT compared to control groups on changes in weight and changes in all EBTs apart from external eating. We found no evidence for differential effectiveness of ACT-based interventions by baseline EBTs when examined as continuous variables. To explore potential non-linear moderating effects, we also examined interactions with EBTs categorised into *“low”, “medium”* and *“high”* levels. While we found no evidence of a moderating effect of low versus medium levels of EBTs, we did find evidence to suggest an increased benefit of ACT over control for participants with medium compared to high levels of internal disinhibition at 12 months post-intervention. We observed similar moderating effects in several sensitivity analyses of both internal disinhibition and emotional eating at intervention end, again suggesting greater intervention benefits for participants with medium levels of these traits.

The theoretical plausibility of these interaction effects is strengthened by the fact that emotional eating and internal disinhibition are correlated traits sharing overlapping questionnaire items [61]. Findings are also in line with a qualitative study on emotional eating in an ACT-based intervention, where participants found the intervention helpful for managing their emotional eating, but those with complex emotional eating experiences expressed a need for more intensive psychological support [29]. The reduced efficacy of treatment for people with high levels of internal disinhibition and emotional eating could be explained by co-occurring psychological conditions, including mental health and eating disorder comorbidities. For example, higher levels of emotional eating and internal disinhibition have been found to be associated with Depression [15, 62–66], Post Traumatic Stress Disorder (PTSD) or trauma [67–69], and Binge Eating or Binge Eating Disorder (BED) [70–72]. In line with this notion, a recent review and aggregate-data meta-regression by Forman et al. [73] found that while BWMIs were generally effective for the full participating sample, BWMIs were less effective for those with comorbid BED compared to those without. Individuals with high internal disinhibition and emotional eating and associated symptoms of diagnosed or undiagnosed mental health comorbidities may require more intensive psychological support by licenced therapists. However, in practice, such at-risk patients are often excluded from weight management services and face long waitlists before they can access appropriate support [74]. It is thus crucial to identify ways to ensure that people with high levels of internal disinhibition or emotional eating receive adequate treatment for both weight loss and potential co-occurring mental health difficulties in the future.

Although the direction of effect for the moderating role of medium compared to high levels of emotional eating and internal disinhibition was consistent across sensitivity analyses, there were differences in the size of the effects and the confidence intervals. This indicates that our findings may be sensitive to different participant and intervention characteristics, and their robustness across contexts remains to be evaluated more closely by future research. In particular, we found the size of the interaction estimate for emotional eating was noticeably small in trials with minimal comparison groups. It would be interesting for future research to confirm whether the observed advantages of ACT for individuals with moderate levels of emotional eating and internal disinhibition are only discernible when ACT is compared to other active treatments rather than to no treatment at all. This would provide additional insights into the suitability of these EBTs as targets for precision treatment approaches using ACT.

We found no evidence of an effect for EBTs as continuous moderators nor when comparing *“low”* and *“medium”* levels of EBTs. However, *“no evidence of an effect”* does not equate to *“evidence of no effect”* [75]. Potential interaction effects may have been too small to be detected or there may have been too much variation across trials. Additionally, the linear interaction structure of continuous effects may not have reflected the *“true”* shape of effects, and similarly the choice of cut-off scores for *“low”* and *“medium”* levels of EBTs may have influenced results [75]. The only previous studies that we are aware of that examined moderating effects of ACT-based interventions and EBTs on weight loss were included in this IPD sample, thus providing limited additional insights [31, 32].

For external eating, i.e. the tendency to overeat in response to environmental cues, we observed that most values contained in the confidence interval were compatible with greater intervention benefit for lower levels of the trait. However, the confidence interval still narrowly crossed zero, indicating that the *“true”* effect could also be zero. Nonetheless, at first glance, the direction of the interaction estimate may seem unexpected. As greater external eating is associated with weight gain, one might anticipate that interventions targeting external eating would be more effective for individuals with higher initial levels of this trait [76, 77]. However, this expectation assumes that external eating is successfully reduced during the intervention. Since we found no evidence that ACT-based interventions changed external eating, this could explain the observed direction of the interaction estimate. ACT-based interventions may not sufficiently address external eating, and, as a result, [76, 77] individuals with higher levels of this trait may continue to struggle with external eating and experience less weight loss compared to those with lower external eating scores [76, 77]. It would be interesting to further explore the role of external eating in future trials, particularly in BWMIs showing a significant effect on changes in external eating.

### Strengths and limitations

A key strength of this study was the use of IPD identified from a comprehensive and systematic search of eight relevant databases. This allowed us to generate the largest database of published and unpublished ACT-based trials for the exploration of effect modifiers to date, with all except one eligible study included. Additionally, we derived within-trial interactions to investigate the moderating role of EBTs, which mitigates the risk of aggregation bias [41].

However, our findings are constrained by certain methodological shortcomings as well as limitations related to the sample obtained. Included trials were mostly conducted in the USA or other high-income countries, and most participants were women (81%), limiting the generalisability of findings to other populations. As is typical for secondary data analyses, we were restricted to the variables available in each trial, preventing the exploration and adjustment of additional factors that could have impacted moderating effects. For example, Forman et al. previously found that therapist expertise moderated outcomes [31].

While we followed the latest guidance on analysis of IPD [41] and collected a total of 2535 observations, the sample sizes of individual trials were small, and models were complex. Additionally, our choice of cut-off scores for the investigation of moderating effects of categorical EBTs (using the tertiles of the pooled sample) may be considered arbitrary [41]. Normative scores for a more meaningful categorisation of EBTs into low, medium, and high values remain to be provided by future research. Additionally, the self-reported nature of EBTs measures and associated considerations regarding their ecological validity should be taken into account when interpreting findings [78, 79].

### Directions for future research and practice

Large confirmatory trials are needed to replicate hypothesised moderating effects using simpler statistical models that do not require a clustered structure. These trials could also include a broader range of bio-psycho-social contributing factors, such as therapist expertise, to enable a more comprehensive exploration of moderating effects. In order to continue to provide high-quality evidence using IPD meta-analyses, future trials should facilitate data sharing by publishing comprehensive data dictionaries or by making their de-identified IPD available in public data repositories. Future research could also explore how EBTs interact to impact weight loss outcomes and attempt to derive meaningful cut-off scores on EBT scales that can aid intervention allocation. Lastly, research may strive to identify adequate care for people with high emotional eating or internal disinhibition scores.

### Conclusion

While, on average, our results suggest that ACT-based BWMIs are a suitable option for adults seeking weight management support, we found evidence to suggest a greater intervention benefit for participants with medium compared to high levels of emotional eating and internal disinhibition. Although confirmatory trials are needed before firm conclusions can be drawn, our findings tentatively support the feasibility of EBT-based precision approaches targeting emotional eating and internal disinhibition with ACT. Future research should aim to ensure adequate support is identified for those with high levels of emotional eating and internal disinhibition.

## Supplementary information

Supplementary Material

## Data Availability

This study used secondary data obtained from original investigators under strict data sharing agreements. Thus, any request for data should be made to original owners of data.

## Code availability

Analysis code can be made available upon request to the corresponding author.
