# Supplementary material for: Individual participant data meta-analysis of eating behaviour traits as effect modifiers in acceptance and commitment therapy-based weight management interventions
Source: Int J Obes (Lond). 2025 Apr 10;49(6):1142–52. doi: 10.1038/s41366-025-01759-9 (PMC12158767; doi:10.1038/s41366-025-01759-9)
Supplement: Supplementary file 1 — Supplementary Material [file 41366_2025_1759_MOESM1_ESM.pdf]

Individual Participant Data Meta-analysis of Eating Behaviour Traits as Effect Modifiers in Acceptance and Commitment Therapy-based Weight Management Interventions

[SUPPLEMENTARY MATERIAL]

Table of Contents

1.0 PRISMA-IPD Checklist 5

2.0 PICO(S) Terms 9

3.0 Full electronic search strategy 10

3.1 MEDLINE via Ovid 10

3.2 EMBASE via Ovid 10

3.3 CENTRAL via Cochrane 10

3.4 ASSIA via ProQuest 11

3.5 WEB OF SCIENCE via web of science 12

3.6 CINAHL via EBSCOhost 12

3.7 AMED via Ovid 12

3.8 PSYCINFO via ProQuest 13

4.0 Data extraction form 14

5.0 Reasons for non-provision of IPD 17

6.0 Risk of Bias assessments 18

6.1 RoB assessment of studies providing IPD 18

6.2 RoB assessment of studies not providing IPD 18

7.0 Study characteristics 19

7.1 Study characteristics of studies providing IPD 19

7.2 Study characteristics of studies not providing IPD 46

8.0 Number of excluded observations and reasons 48

9.0 Participant Characteristics of included studies 50

10.0 Baseline EBTs of included studies 53

11.0 Cut-off scores for EBT Strata 55

12.0 Observed relationship of EBT scores and percent weight change 56

13.0 Intervention effects on weight change 62

14.0 Intervention effects on changes in eating behaviour traits 62

15.0 Sensitivity analyses 63

16.0 References Supplementary Material 70

Tables

Table SM 1-1: PRISMA-IPD Checklist indicating the manuscripts fulfillment of outlined criteria 5
Table SM 2-1: PICO(S) terms indicating eligibility criteria guiding the identification and inclusion of studies 9
Table SM 5-1: Reasons for not providing IPD and exclusion 17
Table SM 7-1: Detailed characteristics of included studies that provided IPD as extracted from published manuscripts 19
Table SM 7-2: Detailed characteristics of eligible studies that were excluded and did not provide IPD 46
Table SM 8-1: Number of excluded observations and reasons for exclusion as indicated by original study authors 48
Table SM 9-1: Participant characteristics in included studies as derived from individual participant data 50
Table SM 9-2 Participant weight change in included studies as derived from individual participant data 51
Table SM 10-1: Baseline eating behaviour traits in included studies as derived from individual participant data 53
Table SM 11-1 Cut-off scores for 'Low', 'Medium' and 'High' Eating Behaviour Trait Strata based on Sample Tertiles 55
Table SM 13-1 The effect of intervention on percentage weight change at end of intervention, 6 – and 12 month follow-up 62
Table SM 14-1 The effect of intervention on changes in eating behaviour traits from baseline to end of intervention 62
Table SM 15-1 Sensitivity analyses of intervention and interaction effects for emotional eating at end of intervention 63
Table SM 15-2 Sensitivity analyses of intervention and interaction effects for external eating at end of intervention 65
Table SM 15-3 Sensitivity analyses of intervention and interaction effects for internal disinhibition at end of intervention 66
Table SM 15-4 Sensitivity analyses of intervention and interaction effects for restraint at end of intervention 67
Table SM 15-5 Sensitivity analyses of intervention and interaction effects for uncontrolled eating at end of intervention 69

Figures

Figure SM 6-1: Risk of Bias assessment of included studies using a modified version of the Cochrane Risk of Bias tool 2 (RoB2) 18
Figure SM 6-2: Risk of Bias assessment of eligible studies that did not provide IPD using a modified version of the Cochrane Risk of Bias tool 2 (RoB2) 18
Figure SM 12-1 Eating Behaviour Trait scores against percentage weight change in the overall sample 56
Figure SM 12-2 Eating Behaviour Trait scores against percentage weight change in trials with a standard behavioural control group 57
Figure SM 12-3 Eating Behaviour Trait scores against percentage weight change in trials with a minimal control group 58

Figure SM 12-4 Eating Behaviour Trait scores against percentage weight change in trials that significantly reduced experiential avoidance 59
Figure SM 12-5 Eating Behaviour Trait scores against percentage weight change in trials that used the Three Factor Eating Questionnaire 60
Figure SM 12-6 Eating Behaviour Trait scores against percentage weight change in participants that attended at least 60% of intervention sessions 61

1.0 PRISMA-IPD Checklist

Table SM 1-1: PRISMA-IPD Checklist indicating the manuscripts fulfillment of outlined criteria

PRISMA-IPD Section/topic	Item No	Checklist item	**Reported on page**
Title			
Title	1	Identify the report as a systematic review and meta-analysis of individual participant data.	1
Abstract			
Structured summary	2	Provide a structured summary including as applicable:	4
		**Background**: state research question and main objectives, with information on participants, interventions, comparators and outcomes.	
		**Methods**: report eligibility criteria; data sources including dates of last bibliographic search or elicitation, noting that IPD were sought; methods of assessing risk of bias.	
		**Results**: provide number and type of studies and participants identified and number (%) obtained; summary effect estimates for main outcomes (benefits and harms) with confidence intervals and measures of statistical heterogeneity. Describe the direction and size of summary effects in terms meaningful to those who would put findings into practice.	
		**Discussion:** state main strengths and limitations of the evidence, general interpretation of the results and any important implications.	
		**Other:** report primary funding source, registration number and registry name for the systematic review and IPD meta-analysis.	
Introduction			
Rationale	3	Describe the rationale for the review in the context of what is already known.	5
Objectives	4	Provide an explicit statement of the questions being addressed with reference, as applicable, to participants, interventions, comparisons, outcomes and study design (PICOS). Include any hypotheses that relate to particular types of participant-level subgroups.	6
Methods			
Protocol and registration	5	Indicate if a protocol exists and where it can be accessed. If available, provide registration information including registration number and registry name. Provide publication details, if applicable.	7
Eligibility criteria	6	Specify inclusion and exclusion criteria including those relating to participants, interventions, comparisons, outcomes, study design and characteristics (e.g. years when conducted, required minimum follow-up). Note whether these were applied at the study or individual level i.e. whether eligible participants were included (and ineligible participants excluded) from a study that included a wider population than specified by the review inclusion criteria. The rationale for criteria should be stated.	7-8
Identifying studies - information sources	7	Describe all methods of identifying published and unpublished studies including, as applicable: which bibliographic databases were searched with dates of coverage; details of any hand searching including of conference proceedings; use of study registers and agency or company databases; contact with the original research team and experts in the field; open adverts and surveys. Give the date of last search or elicitation.	8

Identifying studies - search	8	Present the full electronic search strategy for at least one database, including any limits used, such that it could be repeated.	SM Section 3.0
Study selection processes	9	State the process for determining which studies were eligible for inclusion.	8
Data collection processes	10	Describe how IPD were requested, collected and managed, including any processes for querying and confirming data with investigators. If IPD were not sought from any eligible study, the reason for this should be stated (for each such study).	8-10
		If applicable, describe how any studies for which IPD were not available were dealt with. This should include whether, how and what aggregate data were sought or extracted from study reports and publications (such as extracting data independently in duplicate) and any processes for obtaining and confirming these data with investigators.	
Data items	11	Describe how the information and variables to be collected were chosen. List and define all study level and participant level data that were sought, including baseline and follow-up information. If applicable, describe methods of standardising or translating variables within the IPD datasets to ensure common scales or measurements across studies.	7-10
IPD integrity	A1	Describe what aspects of IPD were subject to data checking (such as sequence generation, data consistency and completeness, baseline imbalance) and how this was done.	9
Risk of bias assessment in individual studies.	12	Describe methods used to assess risk of bias in the individual studies and whether this was applied separately for each outcome. If applicable, describe how findings of IPD checking were used to inform the assessment. Report if and how risk of bias assessment was used in any data synthesis.	10
Specification of outcomes and effect measures	13	State all treatment comparisons of interests. State all outcomes addressed and define them in detail. State whether they were pre-specified for the review and, if applicable, whether they were primary/main or secondary/additional outcomes. Give the principal measures of effect (such as risk ratio, hazard ratio, difference in means) used for each outcome.	10 -12
Synthesis methods	14	Describe the meta-analysis methods used to synthesise IPD. Specify any statistical methods and models used. Issues should include (but are not restricted to):<br>• Use of a one-stage or two-stage approach.<br>• How effect estimates were generated separately within each study and combined across studies (where applicable).<br>• Specification of one-stage models (where applicable) including how clustering of patients within studies was accounted for.<br>• Use of fixed or random effects models and any other model assumptions, such as proportional hazards.<br>• How (summary) survival curves were generated (where applicable).<br>• Methods for quantifying statistical heterogeneity (such as I^2 and τ^2).<br>• How studies providing IPD and not providing IPD were analysed together (where applicable).<br>• How missing data within the IPD were dealt with (where applicable).	10 -12
Exploration of variation in effects	A2	If applicable, describe any methods used to explore variation in effects by study or participant level characteristics (such as estimation of interactions between effect and covariates). State all participant-level characteristics	10 -12

		that were analysed as potential effect modifiers, and whether these were pre-specified.	
Risk of bias across studies	15	Specify any assessment of risk of bias relating to the accumulated body of evidence, including any pertaining to not obtaining IPD for particular studies, outcomes or other variables.	10
Additional analyses	16	Describe methods of any additional analyses, including sensitivity analyses. State which of these were pre-specified.	12
**Results**			
Study selection and IPD obtained	17	Give numbers of studies screened, assessed for eligibility, and included in the systematic review with reasons for exclusions at each stage. Indicate the number of studies and participants for which IPD were sought and for which IPD were obtained. For those studies where IPD were not available, give the numbers of studies and participants for which aggregate data were available. Report reasons for non-availability of IPD. Include a flow diagram.	13-14
Study characteristics	18	For each study, present information on key study and participant characteristics (such as description of interventions, numbers of participants, demographic data, unavailability of outcomes, funding source, and if applicable duration of follow-up). Provide (main) citations for each study. Where applicable, also report similar study characteristics for any studies not providing IPD.	13-14
IPD integrity	A3	Report any important issues identified in checking IPD or state that there were none.	14-15
Risk of bias within studies	19	Present data on risk of bias assessments. If applicable, describe whether data checking led to the up-weighting or down-weighting of these assessments. Consider how any potential bias impacts on the robustness of meta-analysis conclusions.	15
Results of individual studies	20	For each comparison and for each main outcome (benefit or harm), for each individual study report the number of eligible participants for which data were obtained and show simple summary data for each intervention group (including, where applicable, the number of events), effect estimates and confidence intervals. These may be tabulated or included on a forest plot.	Not applicable to one-stage approach
Results of syntheses	21	Present summary effects for each meta-analysis undertaken, including confidence intervals and measures of statistical heterogeneity. State whether the analysis was pre-specified, and report the numbers of studies and participants and, where applicable, the number of events on which it is based.	15 - 17
		When exploring variation in effects due to patient or study characteristics, present summary interaction estimates for each characteristic examined, including confidence intervals and measures of statistical heterogeneity. State whether the analysis was pre-specified. State whether any interaction is consistent across trials.	
		Provide a description of the direction and size of effect in terms meaningful to those who would put findings into practice.	
Risk of bias across studies	22	Present results of any assessment of risk of bias relating to the accumulated body of evidence, including any pertaining to the availability and representativeness of available studies, outcomes or other variables.	15

Additional analyses	23	Give results of any additional analyses (e.g. sensitivity analyses). If applicable, this should also include any analyses that incorporate aggregate data for studies that do not have IPD. If applicable, summarise the main meta-analysis results following the inclusion or exclusion of studies for which IPD were not available.	15-17
Discussion			
Summary of evidence	24	Summarise the main findings, including the strength of evidence for each main outcome.	17-18
Strengths and limitations	25	Discuss any important strengths and limitations of the evidence including the benefits of access to IPD and any limitations arising from IPD that were not available.	20
Conclusions	26	Provide a general interpretation of the findings in the context of other evidence.	21
Implications	A4	Consider relevance to key groups (such as policy makers, service providers and service users). Consider implications for future research.	21
Funding			
Funding	27	Describe sources of funding and other support (such as supply of IPD), and the role in the systematic review of those providing such support.	23

A1 – A3 denote new items that are additional to standard PRISMA items. A4 has been created as a result of re-arranging content of the standard PRISMA statement to suit the way that systematic review IPD meta-analyses are reported.

© Reproduced with permission of the PRISMA IPD Group, which encourages sharing and reuse for non-commercial purposes

2.0 PICO(S) Terms

Table SM 2-1: PICO(S) terms indicating eligibility criteria guiding the identification and inclusion of studies

PICOs	Eligibility
Population	• Adults (aged 18 and older) with a BMI ≥ 25 kg/m2.<br>• Studies were excluded if participants were recruited purely based on having a chronic disease or being pregnant, as were studies where eligible participants resided in institutional settings (e.g. hospital, army barracks).<br>• Studies on children and adolescents were not considered for inclusion to avoid the risk of increasing heterogeneity in interventions serving different target populations.
Intervention	• Interventions with the primary goal of weight loss or weight loss maintenance that report incorporating strategies based on ACT.<br>• ACT-based interventions from different contexts were eligible (e.g. online, in person, health care setting, commercial), and they were eligible either as standalone treatment or as part of a wider weight management intervention.
Comparison	• Inactive/ wait-list control, minimal intervention (e.g. leaflet, brief advice), or an active standard behavioural weight management control
Outcomes	• Outcome: Weight assessed at both baseline and end of intervention. Additionally, a follow-up point of at least 3-months post-baseline had to be available.<br>• Effect modifiers of interest: EBTs assessed at baseline. Eligible EBTs were emotional eating, uncontrolled eating, disinhibition (general disinhibition, internal disinhibition, external disinhibition), external eating and restraint (general restraint, flexible restraint, rigid restraint). Disordered eating was excluded.
Study Design	• RCTs and cluster-RCTs

3.0 Full electronic search strategy

3.1 MEDLINE via Ovid

**1**	exp Obesity/ OR exp Overweight/ OR exp Body Weight/ OR exp Body Mass Index/ OR exp Waist Circumference/ OR exp Feeding Behavior/ OR exp Body Weight Changes/ OR exp Caloric Restriction/ OR exp Weight Loss/ OR obes*.mp OR (overweight or over-weight).mp OR (weight adj3 (body or chang* or loss* or maint* or manag* or control* or reduct*)).mp OR (food adj3 (intake or habit*)).mp OR (body mass index or bmi).mp OR body adj3 mass.mp OR (calori* adj3 (restrict* or restrain* or reduc*)).mp OR feeding adj3 behavio*.mp OR (diet* adj3 (restrict* or restrain* or reduc*)).mp OR (waist* adj3 circumferenc*).mp
**2**	"Acceptance and Commitment Therapy"/ OR (acceptance* adj3 (commit* or mind* or base* or focus* or intervention* or therap* or treat*)).mp
**3**	1 AND 2
**4**	Limit 3 to dt=20190925-20220620
**5**	Limit 3 to rd=20190925-20220620
**6**	4 OR 5

3.2 EMBASE via Ovid

exp Obesity/ OR exp Body Weight/ OR exp Body Mass/ OR exp Waist Circumference/ OR exp Feeding Behavior/ OR exp Caloric Restriction/ OR exp Weight Reduction/ OR obes*.mp OR (overweight or over-weight).mp OR (weight adj3 (body or chang* or loss* or maint* or manag* or control* or reduct*)).mp OR (food adj3 (intake or habit*)).mp OR (body mass index or bmi).mp OR (body adj3 mass).mp OR (calori* adj3 (restrict* or restrain* or reduc*)).mp OR (feeding adj3 behavio*).mp OR (diet* adj3 (restrict* or restrain* or reduc*)).mp OR (waist* adj3 circumferenc*).mp

AND

"acceptance and commitment therapy"/ OR (acceptance* adj3 (commit* or mind* or base* or focus* or intervention* or therap* or treat*)).mp

Limit NUM to dd=20190925-20220620

Limit NUM to rd=20190925-20220620

NUM or NUM

3.3 CENTRAL via Cochrane

#1	MeSH descriptor: [Obesity] explode all trees	15762
#2	MeSH descriptor: [Overweight] explode all trees	18878
#3	MeSH descriptor: [Body Weight] explode all trees	31138
#4	MeSH descriptor: [Body Mass Index] explode all trees	10927
#5	MeSH descriptor: [Waist Circumference] explode all trees	1143

#6	MeSH descriptor: [Feeding Behavior] explode all trees	9769
#7	MeSH descriptor: [Body Weight Changes] explode all trees	9695
#8	MeSH descriptor: [Caloric Restriction] explode all trees	941
#9	MeSH descriptor: [Weight Loss] explode all trees	7104
#10	obes* in All Text	51603
#11	(overweight or over-weight)	19265
#12	(weight near/3 (body or chang* or loss* or maint* or manag* or control* or reduct*))	75571
#13	(food near/3 (intake or habit*))	11473
#14	(body mass index or bmi)	74353
#15	(body near/3 mass)	64975
#16	(calori* near/3 (restrict* or restrain* or reduc*))	3305
#17	feeding near/3 behavio*	5662
#18	(diet* near/3 (restrict* or restrain* or reduc*))	15513
#19	(waist* near/3 circumferenc*)	10789
#20	#1 or #2 or #3 or #4 or #5 or #6 or #7 or #8 or #9 or #10 or #11 or #12 or #13 or #14 or #15 or #16 or #17 or #18 or #19	171531
#21	MeSH descriptor: [Acceptance and Commitment Therapy] explode all trees	281
#22	(acceptance* near/3 (commit* or mind* or base* or focus* or intervention* or therap* or treat*))	2898
#23	#21 or #22	2898
#24	#20 and #23	324

Apply limits > Select limits > Run search

3.4 ASSIA via ProQuest

MAINSUBJECT.EXACT.EXPLODE("Obesity") OR MAINSUBJECT.EXACT.EXPLODE("Body weight") OR MAINSUBJECT.EXACT.EXPLODE("Body Mass Index") OR MAINSUBJECT.EXACT.EXPLODE("Feeding patterns") OR MAINSUBJECT.EXACT.EXPLODE("Caloric intake") OR obes* OR overweight OR over-weight OR (weight NEAR/3 (body or chang* or loss* or maint* or manag* or control* or reduct*)) OR (food NEAR/3 (intake or habit*)) OR ("body mass index" or bmi) OR (body NEAR/3 mass) OR (calori* NEAR/3 (restrict* or restrain* or reduc*)) OR (feeding NEAR/3 (pattern* or behavio*)) OR (diet* NEAR/3 (restrict* or restrain* or reduc*)) OR (waist* NEAR/3 circumferenc*)

AND

acceptance* NEAR/3 (commit* or mind* or base* or focus* or intervention* or therap* or treat*)

Limit by publication date (2019-09-25 to 2022-06-21)

3.5 WEB OF SCIENCE via web of science

(TS=(obes*) OR TS=(overweight or over-weight) OR TS=(weight NEAR/3 (body or chang* or loss* or maint* or manag* or control* or reduct*)) OR TS=(food NEAR/3 (intake or habit*)) OR TS=(“body mass index” or bmi) OR TS=(body NEAR/3 mass) OR TS=(calori* NEAR/3 (restrict* or restrain* or reduc*)) OR TS=(feeding NEAR/3 behavio*) OR TS=(diet* NEAR/3 (restrict* or restrain* or reduc*)) OR TS=(waist* NEAR/3 circumferenc*)) AND (TS=(acceptance* NEAR/3 (commit* or mind* or base* or focus* or intervention* or therap* or treat*)))

Refined by: PUBLICATION date (2019-09-25 to 2022-06-20)

3.6 CINAHL via EBSCOhost

((MH "Obesity+") OR (MH "Body Weight+") OR (MH "Eating Behavior+") OR (MH "Body Weight Changes+") OR (MH "Weight Loss+") OR (MH "Body Mass Index") OR (MH "Waist Circumference") OR (MH "Weight Reduction Programs") OR (TX obes*) OR (TX (overweight or over-weight)) OR (TX (weight N3 (body or chang* or loss* or maint* or manag* or control* or reduct*))) OR (TX (food N3 (intake or habit*))) OR (TX (body mass index or bmi)) OR (TX (body N3 mass)) OR (TX (calori* N3 (restrict* or restrain* or reduc*))) OR (TX (feeding N3 behavio*)) OR (TX (diet* N3 (restrict* or restrain* or reduc*))) OR (TX (waist* N3 circumferenc*)))

AND

((MH "Acceptance and Commitment Therapy") OR (TX (acceptance* N3 (commit* or mind* or base* or focus* or intervention* or therap* or treat*))))

Limiters - Published Date: 20190801-20220631

3.7 AMED via Ovid

exp Obesity/ or exp Body Weight/ or exp Body Mass Index/ or exp Weight Loss/ or obes*.mp or (overweight or over-weight).mp or (weight adj3 (body or chang* or loss* or maint* or manag* or control* or reduct*)).mp or (food adj3 (intake or habit*)).mp or (body mass index or bmi).mp or body adj3 mass.mp or (calori* adj3 (restrict* or restrain* or reduc*)).mp or feeding adj3 behavio*.mp or (diet* adj3 (restrict* or restrain* or reduc*)).mp or (waist* adj3 circumferenc*).mp

AND

(acceptance* adj3 (commit* or mind* or base* or focus* or intervention* or therap* or treat*)).mp

Limit NUM to 2018-Current

3.8 PSYCINFO via ProQuest

MAINSUBJECT.EXACT.EXPLODE("Obesity") OR MAINSUBJECT.EXACT.EXPLODE("Body Weight") OR MAINSUBJECT.EXACT.EXPLODE("Body Mass Index") OR MAINSUBJECT.EXACT.EXPLODE("Eating Behavior") OR MAINSUBJECT.EXACT.EXPLODE("Food Intake") OR

MAINSUBJECT.EXACT.EXPLODE("Diets") OR obes* OR overweight OR over-weight OR (weight NEAR/3 (body or chang* or loss* or maint* or manag* or control* or reduct*)) OR (food NEAR/3 (intake or habit*)) OR ("body mass index" or bmi) OR (body NEAR/3 mass) OR (calori* NEAR/3 (restrict* or restrain* or reduc*)) OR (feeding NEAR/3 (pattern* or behavio*)) OR (diet* NEAR/3 (restrict* or restrain* or reduc*)) OR (waist* NEAR/3 circumferenc*)

AND

MAINSUBJECT.EXACT.EXPLODE("Acceptance and Commitment Therapy") OR acceptance* NEAR/3 (commit* or mind* or base* or focus* or intervention* or therap* or treat*)

Filtered by 2019-09-25 - 2022-06-21

4.0 Data extraction form

Study information

ID of data extractor	
Covidence ID	
References (Author, Publication year, Titles)	
Trial ID (if provided)	

Study characteristics

Methods

Design (e.g. randomised or not, number of trial arms, pilot or not)		
Setting (e.g. clinical, commercial, workplace, etc.)		
Country		
Inclusion/exclusion criteria	Inclusion criteria	
	Exclusion criteria	
Inclusion based on specific population characteristic? (e.g. pre-existing condition, gender, ethnicity)		
Funding statement (copy verbatim)		
Declarations of interest statement (copy verbatim)		
Any other notes to be included in characteristics of included studies table		

Participants (at baseline)

Total N randomized	
N per arm/group (where relevant)	
Total % Female	
Total mean age	
Total mean baseline BMI	
Total Ethnicity	
Total Education	
Total Socioeconomic status	
Total mean baseline EBT (rename and add as appropriate)	

Interventions (where multiple arms, copy and paste for each arm)

Any shared aspects between intervention and control groups	Target behaviour (e.g. physical activity or nutrition)	
	Mode of Delivery (e.g. group vs individual, online vs in person)	
	Duration & Frequency & Intensity (Dose)	
	Shared Intervention Content/ Components	
Comparison Arm *(rename as needed)*	Control type (e.g. waitlist / inactive, minimal intervention, usual care, standard behavioural intervention)	
	Target behaviour (e.g. physical activity or nutrition)	
	Mode of Delivery (e.g. group vs individual, online vs in person)	
	Duration & Frequency & Intensity (Dose)	
	Intervention type (e.g. CBT) and components, if applicable	
	Other intervention details	
Intervention Arm *(rename as needed)*	Target behaviour (e.g. physical activity or nutrition)	

	Mode of Delivery (e.g. group vs individual, online vs in person)	
	Duration & Frequency & Intensity (Dose)	
	ACT components (add key terms – see table on page 4)	
	Any other behaviour change strategies or intervention components	
	Other intervention details	

Moderators/Outcomes

How EBTs were measured/defined (which questionnaire was used)	
When EBTs were measured	
How weight outcome was measured (e.g. self-report vs objective etc.)	
When outcome was measured	

Outcomes (Only extract if IPD is not provided)

	Effect measure (replace as appropriate)	N (describe if taken from anywhere else other than results table)
**EBT** (mean, SD or change score) at end of intervention (please list all availiable EBTs)		
**EBT** (mean, SD or change score) at follow-up		
**Weight** (please specifiy what weight outcome used) at baseline (mean, SD)		
**Weight** (please specifiy what weight outcome used) at end of intervention (mean, SD or change score)		
**Weight** (please specifiy what weight outcome used) at follow-up (mean, SD or change score)		

5.0 Reasons for non-provision of IPD

Table SM 5-1: Reasons for not providing IPD and exclusion

Study	Reason	Citation(s)
Afari et al. (2019)	Lost contact [a]	[1–3]

[a] refers to not receiving a reply after reaching out via email on three consecutive occasions

6.0 Risk of Bias assessments

6.1 RoB assessment of studies providing IPD

Figure SM 6-1: Risk of Bias assessment of included studies using a modified version of the Cochrane Risk of Bias tool 2 (RoB2)

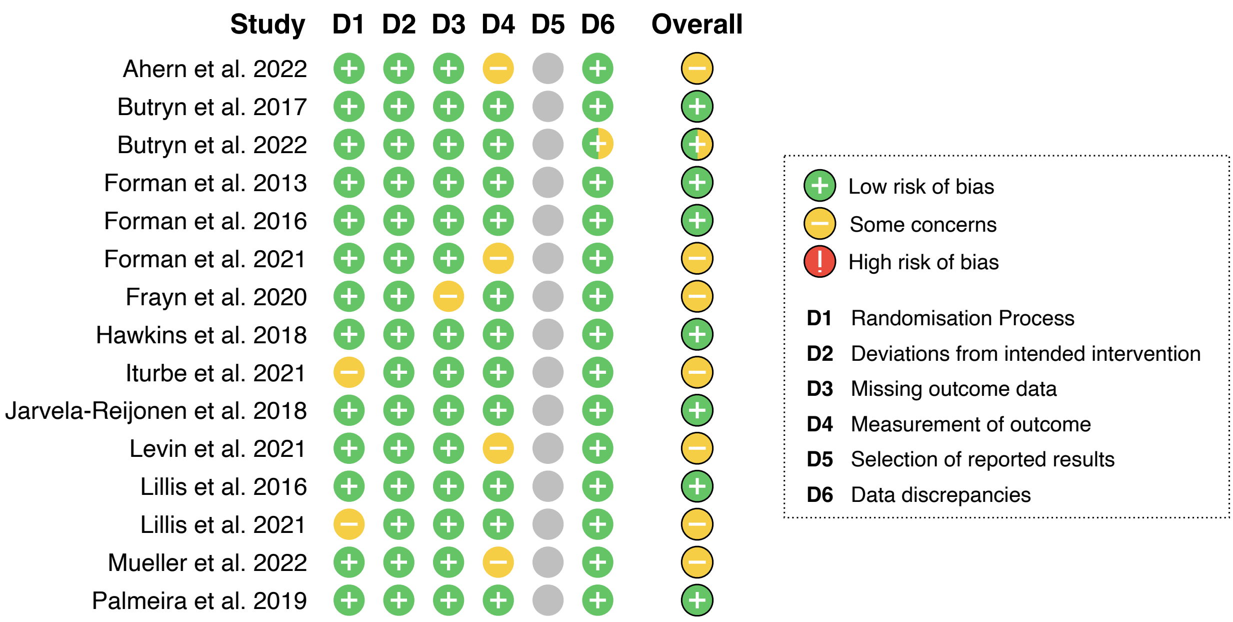

Note: Butryn 2022 was classified as some concerns only for the emotional eating outcome due to data discrepancies (D6)

6.2 RoB assessment of studies not providing IPD

Figure SM 6-2: Risk of Bias assessment of eligible studies that did not provide IPD using a modified version of the Cochrane Risk of Bias tool 2 (RoB2)

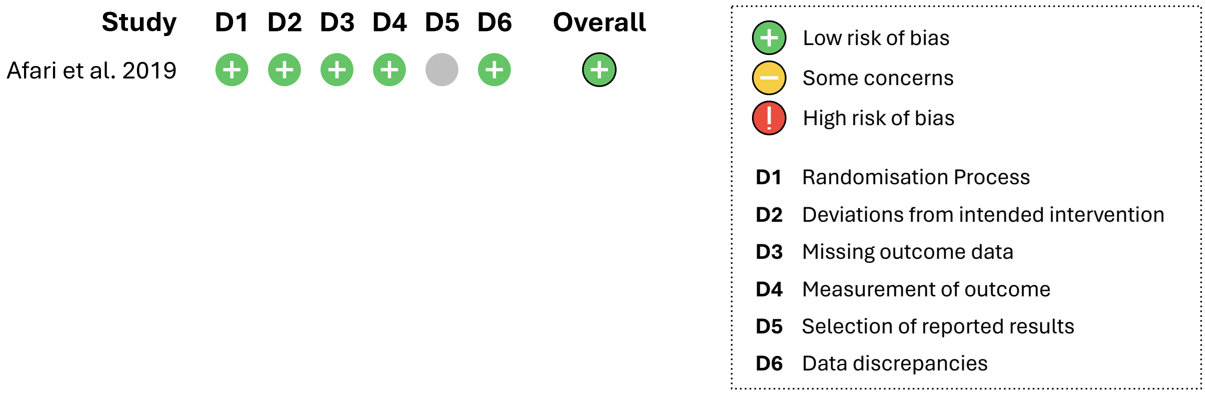

7.0 Study characteristics

7.1 Study characteristics of studies providing IPD

Table SM 7-1: Detailed characteristics of included studies that provided IPD as extracted from published manuscripts

Study	Methods	Participant characteristics	Intervention and comparators	Exposures, outcomes and time points
Ahern et al. (2022) [4]	Study design:<br><br>Ongoing two-arm pilot RCT<br><br>Country:<br><br>UK<br><br>Eligibility:<br><br>Adults (≥18y) who completed a behavioural weight loss intervention lasting at least 12 weeks in the last 3 months	Sex: 84% female<br><br>Age in years[1]: 48.0 (14.1)<br><br>BMI (kg/m^2)[1]: 38.9 (8.7)<br><br>Ethnicity: 95% White<br><br>Education: 51% Below post-secondary<br><br>Income: 48% have to be careful about money	Comparator (N = 20):<br><br>• **Type:** Usual care<br>• **Aim:** Weight loss maintenance<br>• **Intensity and delivery:** one-off email containing a leaflet<br>• **Content:** leaflet about how to make a personalised weight loss maintenance plan<br><br>Intervention (N = 41):<br><br>• **Aim:** Weight loss maintenance<br>• **Intensity and delivery:** 14 online modules of 30 to 60 minutes expected completion duration (13 weekly modules, then 4 weeks break until last module) + 4 scheduled telephone support calls from a trained non-specialist "SWiM coach"	EBT Measure(s):<br><br>• Emotional eating (TFEQ-21)<br>• Uncontrolled eating (TFEQ-21)<br>• Restraint (TFEQ-21)<br>Outcome Measure(s):<br><br>• Self-reported weight<br>Assessment time points:<br><br>• Baseline<br>• 6 months

			at weeks 1, 3, 8, and 17 + 3 optional calls to be used at any time<br>● **Content:** Guided self-help ACT-based weight management intervention with behavioural strategies including goal setting, self-monitoring, planning, social support, strategies to prevent lapses<br>● **ACT components:**<br>○ Acceptance<br>○ Willingness<br>○ Value clarification<br>○ Cognitive defusion<br>○ Comitted Action<br>○ Present moment awareness	
Butryn et al. 2017[5]	Study design:<br>Three-arm RCT (comparison arm merged for meta-analyses)<br>Country:<br>USA<br>Eligibility:<br>Adults (≥18y ≤70y) with a BMI between 27 and 45kg/m^2	Sex: 78.8% female<br>Age in years[1]:<br>SBT = 53.02 (9.32)<br>SBT + E = 53.41 (10.28)<br>SBT + EA = 53.23 (9.43)<br>BMI (kg/m^2)[1]:<br>SBT = 34.96 (5.19)<br>SBT + E = 35.38 (5.17)<br>SBT + EA = 35.23 (4.64)<br>Ethnicity:	Comparators:<br>(1) SBT (N = 88)<br>● **Type:** Standard behavioural treatment (SBT)<br>● **Aim:** Weight loss/ diet and physical activity<br>● **Intensity and delivery:** 26 sessions over 52 weeks with groups of 10-14 participants of 75 mins duration, delivered by clinicians with a masters or doctoral degraa in psychology and training in conducting behavioural weight loss interventions	EBT Measure(s):<br>● Emotional Eating (TFEQ-R18)<br>● Uncontrolled Eating (TFEQ-R18)<br>● Restraint (TFEQ-R18)<br>● Disinhibition (TFEQ-51)<br>Outcome Measure(s):<br>● Objectively measured weight<br>Assessment time points:

		SBT:<br><br>Black or African-American = 27%<br><br>White = 67%<br><br>Hispanic/Latino = 8%<br><br>SBT + E:<br><br>Black or African-American = 29%<br><br>White = 68%<br><br>Hispanic/ Latino = 4%<br><br>SBT + EA:<br><br>Black or African-American = 31%<br><br>White = 63%<br><br>Hispanic/Latino = 12%<br><br><br>Education:<br><br>SBT:<br><br>High school or lower = 6%<br><br>Associates degree = 16%<br><br>Bachelors degree = 36%<br><br>Graduate or professional degree = 42%	● **Content:** SBT adapted from the Look AHEAD and the Diabetes Prevention Program protocols, including strategies such as self-monitoring, goal setting, problem solving, identifying triggers, developing social support, relapse prevention<br><br>(2) SBT + E (N = 93)<br><br>● **Type:** SBT + content on the home environment<br>● **Aim:**<br>● **Intensity and delivery:** As above<br>● **Content:** SBT as above, with the addition of strategies including modifying the availability of foods to reduce foods promoting overconsumption and increase foods facilitating weight management, adapting home environment to increase cues of physical activity<br><br>Intervention (N = 102):<br><br>● **Aim:**<br>● **Intensity and delivery:** As above<br>● **Content:** SBT + E as above, with the addition of ACT content<br>● **ACT components:**<br>○ Acceptance	● 6 months (mid-treatment)<br>● 12 months (end of intervention)<br>● 18 months (6-month follow-up)<br>● 24 months (12-month follow-up)

		SBT + E:<br><br>High school or lower = 6%<br><br>Associates degree = 21%<br><br>Bachelors degree = 23%<br><br>Graduate or professional degree = 50%<br><br>SBT + EA:<br><br>High school or lower = 7%<br><br>Associates degree = 12%<br><br>Bachelors degree = 46%<br><br>Graduate or professional degree = 35%<br><br>Income: not reported	○ Willingness<br>○ Values Clarification<br>○ Committed Action	
Butryn et al. (2022) [6–8]	Study design:<br><br>Three-arm RCT<br><br>Country:	Sex: 78.1% female<br><br>Age in years[1]: 52.72 (10.35)<br><br>BMI (kg/m^2)[1]: 35.14 (4.76)	In phase 1, all participants received standard behavioural treatment (SBT) adapted from the Look AHEAD15 and the Diabetes Prevention Program protocols. In phase 2	EBT Measure(s):<br><br>● Emotional Eating (EOQ)<br>● Disinhibition (TFEQ-51)

	USA<br><br><u>Eligibility:</u><br><br>Adults (≥18y ≤70y) with a BMI between 27 and 45kg/m^2	<u>Ethnicity:</u><br><br>Hispanic or Latino = 3.8%<br><br>White or Caucasian = 70.0%<br><br>Black or African American = 25.0%<br><br>More than one race = 2.8%<br><br>Asian = 1.6%<br><br>American Indian or Alaska Native = 0.6%<br><br><u>Education:</u><br><br>High school graduate or less = 5.0%<br><br>Associate's or technical degree or partial college = 17.8%<br><br>Bachelor's degree = 33.1%<br><br>Graduate degree = 44.1%<br><br><u>Income:</u> not reported	they were randomized into one of three conditions:<br><br><u>Comparators:</u><br><br><u>(1) SBT (N = 110)</u><br><br>● **Type:** SBT<br>● **Aim:** Weight loss/ diet and physical activity<br>● **Intensity and delivery:**<br>○ Phase 1: 16 group sessions with 12 participants over a duration of 6 months (8 weekly, 8 bi-weekly), led by counsellors with doctoral level psychology training<br>○ Phase 2: 14 continued group sessions over 12 additional months (18 in total), with 7 weekly, 4 bi-weekly, and 3 sessions in month 12, 15 and 18 plus 3 one-to-one 15-min phone calls<br>● **Content:** continued SBT, including strategies such as self-monitoring, goal setting, problem solving, identifying triggers, developing social support	<u>Outcome Measure(s):</u><br><br>● Objectively measured weight<br><u>Assessment time points:</u><br><br>● Baseline<br>● 6 months (mid-intervention)<br>● 12 months (mid-intervention)<br>● 18 months (end of intervention)<br>● 24 months (6-month follow-up)<br>● 36 months (18-month follow-up)

			(2) SBT + PA (N = 105)<br><br>● **Type:** SBT + Physical Activity Focussed<br>● **Aim:** Weight loss/ diet and physical activity<br>● **Intensity and delivery:** As above<br>● **Content:** focus on physical activity, incorporating techniques from the behaviour change taxonomy<br><br>Intervention (N = 105):<br><br>● **Aim:** Weight loss/ diet and physical activity<br>● **Intensity and delivery:** As above<br>● **Content:** SBT + Acceptance based content, with focus on physical activity<br>● **ACT components:**<br>○ Present Moment Awareness<br>○ Acceptance<br>○ Value clarification<br>○ Committed Action	
Forman et al. (2013) [9,10]	Study design:<br><br>Two-arm RCT<br><br>Country:	Sex: 85.16% female<br><br>Age in years[1]: 45.69 (12.81)<br><br>BMI (kg/m^2)[1]: 34.10 (3.64)	Comparator (N = 54):<br><br>● **Type:** Standard behavioural treatment (SBT) + cognitive behavioural model	EBT Measure(s):<br><br>● Emotional Eating (DEBQ and EES)<br>● Disinhibition (TFEQ-51)

	USA<br><br>Eligibility:<br><br>Adults (≥21y ≤65y) with a BMI between 27 and $40kg/m^2$	Ethnicity:<br><br>Caucasian 62.3%<br><br>African American 24.6%<br><br>Asian: 1.6%<br><br>Hispanic: 3.8%<br><br>Education: not reported<br><br>Income: not reported	● **Aim:** Weight loss/ diet and physical activity<br>● **Intensity and delivery:** 30 group sessions of 75 mins duration, over a timespan of 40 weeks (weekly during weeks 1 to 20, bi-weekly in weeks 21 to 40) led by doctoral students or clinical psychologists with training in conducting behavioural weight loss interventions<br>● **Content:** SBT based on the LEARN and Diabetes Prevention Programm weight loss and maintenance protocols, including nutrition education, and behavioural strategies like self-monitoring, stimulus control, behavioural analysis, relapse prevention, identifying triggers, problem solving, encouraging social support + introduction of the cognitive behavioural model and cognitive restructuring<br><br>Intervention (N = 74):<br><br>● **Aim:** Weight loss/ diet and physical activity<br>● **Intensity and delivery:** As above	Outcome Measure(s):<br><br>● Objectively measures weight<br><br>Assessment time points:<br><br>● Baseline<br>● 10 weeks (early intervention)<br>● 20 weeks (mid-intervention)<br>● 40 weeks (end of intervention<br>● 66 weeks (6-months follow-up)

			• **Content:** SBT components similar to the above (without using the cognitive behavioural model) plus ACT components adapted from treatment descriptions by Hayes<br>• **ACT components:**<br>o Value identification and clarification<br>o Present moment awareness<br>o Observing self/ self as context<br>o Experential and present moment awareness<br>o Defusion (Urge surfing)<br>o Willingness<br>o Acceptance	
Forman et al. (2016) [11–13]	Study design:<br>Two-arm RCT<br>Country:<br>USA<br>Eligibility:<br>Adults (≥18y ≤70y) with a BMI between 27 and 50kg/m^2	Sex: 82.1% female<br>Age in years[1]: 51.64 (0.73)<br>BMI (kg/m^2)[1]: 36.93 (0.42)<br>Ethnicity:<br>Caucasian 70.5%<br>African American: 24.7%<br>Asian: 1.1%<br>Hispanic: 3.7%	Comparator (N = 90):<br>• **Type:** Standard behavioural treatment (SBT) + cognitive behavioural therapy (CBT)<br>• **Aim:** Weight loss/ diet and physical activity<br>• **Intensity and delivery:** 25 group sessions with 10 to 14 participants of 75 mins duration over a period of 12 months (16 weekly, 5 bi-weekly, 2 monthly, 2 bi-mothly), led by doctoral level clinicians with	EBT Measure(s):<br>• Emotional eating (TFEQ-18)<br>• Restraint (TFEQ-18)<br>• Uncontrolled eating (TFEQ-18)<br>• Disinhibition (TFEQ-51)<br>Outcome Measure(s):<br>• Objectively measured weight

		Education: not reported<br><br>Income: not reported	experience delivering behavioural weight management interventions<br>● **Content:** SBT based on the LEARN and Diabetes Prevention Programm weight loss and maintenance protocols, including nutrition and physical activity education, and behavioural strategies like SMART goals, self-monitoring, stimulus control, behavioural analysis, relapse prevention, identifying triggers, problem solving, encouraging social support + CBT including distraction and confrontation, identification of cognitive distortions, cognitive restructuring and building self-esteem<br><br>Intervention (N = 100):<br><br>● **Aim:** Weight loss/ diet and physical activity<br>● **Intensity and delivery:** As above<br>● **Content:** SBT components similar to the above (without CBT) + ACT components. Approximately 85% overlap of treatments<br>● **ACT components:**<br>○ Values clarification<br>○ Acceptance	Assessment time points:<br><br>● Baseline<br>● 6 months (mid-intervention)<br>● 12 months (end of intervention)<br>● 24 months (12-month follow-up)<br>● 36 months (24-month follow-up)

			○ Willingness<br>○ Present moment awareness<br>○ Cognitive defusion<br>○ Committed action	
Forman et al. (2021) [14]	Study design:<br><br>Ongoing 2 x 2 x 2 Factorial Trial<br><br>Country:<br><br>USA<br><br>Eligibility:<br><br>Adults (≥18y ≤70y) with a BMI between 25 and 50kg/m^2	Sex: 86.23% female<br><br>Age in years[1]: 51.84 (10.91)<br><br>BMI (kg/m^2)[1]: 35.79 (5.23)<br><br>Ethnicity: X<br><br>Education: X<br><br>Income: X	Comparator (N = 34):<br><br>• **Type:** Standard Behavioural Treatment (SBT) only<br>• **Aim:** Weight loss/ diet and physical activity<br>• **Intensity and delivery:** 20 remote group sessions (delivered over zoom) of 85 to 130 mins duration (depending on the number of components included) + 3 minute one-to-one consultations about weight trajectory + assignments via Google Classroom<br>• **Content:** SBT adapted from the Diabetes Prevention Programme (DPP) and Look Ahead, including nutrition and physical activity education, self-monitoring, stimulus control, goal setting, problem-solving, barriers to change, improving social support, preventing relapse	EBT Measure(s):<br><br>• Emotional Eating (TFEQ-18 and DEBQ)<br>• Restraint (TFEQ-18)<br>• Uncontrolled eating (TFEQ-18)<br>• Disinhibition (TFEQ-51)<br>Outcome Measure(s):<br><br>• Self-reported weight via wireless scales<br><br>Assessment time points:<br><br>• Baseline<br>• 12 months (end of intervention<br>• 18 months (6-month follow-up)<br>• 24 months (12-month follow-up)<br>• 36 months (24-month follow-up)

			<u>Interventions</u>:<br><br>(1) SBT + Values (N = 32)<br><br>● **Aim:** As above<br>● **Intensity and delivery:** As above<br>● **Content:** SBT as above plus ACT techniques as adapted from the MB-EAT program on values<br>● **ACT components:**<br>○ Value clarification<br>○ Committed Action<br><br>(2) SBT + Awareness (N = 35)<br><br>● **Aim:** As above<br>● **Intensity and delivery:** As above<br>● **Content:** SBT as above plus ACT techniques as adapted from the MB-EAT program on awareness<br>● **ACT components:**<br>○ Present moment awareness<br><br>(3) SBT + Willingness (N = 35)<br><br>● **Aim:** As above<br>● **Intensity and delivery:** As above<br>● **Content:** SBT as above plus ACT techniques as adapted from the MB-EAT program on willingness<br>● **ACT components:**<br>○ Cognitive defusion	

			○ Acceptance<br>○ Committed Action<br><br>(4) SBT + Values + Awareness (N = 38)<br><br>● **Aim:** As above<br>● **Intensity and delivery:** As above<br>● **Content:** SBT as above plus ACT techniques as adapted from the MB-EAT program on values and awareness<br>● **ACT components:**<br>○ Value clarification<br>○ Committed Action<br>○ Present moment awareness<br><br>(5) SBT + Values + Willingness (N = 36)<br><br>● **Aim:** As above<br>● **Intensity and delivery:** As above<br>● **Content:** SBT as above plus ACT techniques as adapted from the MB-EAT program on values and willingness<br>● **ACT components:**<br>○ Value clarification<br>○ Cognitive defusion<br>○ Acceptance<br>○ Committed Action<br><br>(6) SBT + Willingness + Awareness (N = 34)	

			• **Aim:** As above<br>• **Intensity and delivery:** As above<br>• **Content:** SBT as above plus ACT techniques as adapted from the MB-EAT program on willingness and awareness<br>• **ACT components:**<br>○ Cognitive defusion<br>○ Acceptance<br>○ Committed Action<br>○ Present moment awareness<br><br>(7) SBT + Values + Willingness + Awareness (N = 32)<br><br>• **Aim:** As above<br>• **Intensity and delivery:** As above<br>• **Content:** SBT as above plus ACT techniques as adapted from the MB-EAT program on values, willingness and awareness<br>• **ACT components:**<br>○ Value clarification<br>○ Cognitive defusion<br>○ Acceptance<br>○ Committed Action<br>○ Present moment awareness	
Frayn et al. (2020) [15]	Study design:<br><br>Two-arm pilot RCT	Gender: 92% female<br><br>Age in years[1]: 46.91 (14.56)	Comparator (N = 43):<br><br>• **Type:** Usual care<br>• **Aim:** Weight loss	EBT Measure(s):<br><br>• Emotional Eating (DEBQ)

	Country:<br><br>Canada<br><br>Eligibility:<br><br>Adults (≥18y) with a BMI ≥25kg/m^2 and high emotional eating (score of 3.25 or higher on the DEBQ)	Weight (kg^2)[1]: 96.52 (25.61)<br><br>Ethnicity: 59.1% White<br><br>Education: 39.39% some college<br><br>Income: 42.42% annual household income above $40,000	• **Intensity and delivery:** 8 one-to-one sessions delivered by physicians.<br>• **Content:** Diet and exercise counselling and psychoeducation, as routinely performed at the clinic<br><br>Intervention (N = 44):<br><br>• **Aim:** Weight loss and reduction of emotional eating<br>• **Intensity and delivery:** 5-10 minutes added to usual care sessions. Physicians were trained in delivering manualised ACT interventions by a clinical psychology doctoral student. Participants took 7 to 50 weeks to complete the intervention, with an average of 16 weeks<br>• **Content:** Usual care + manualised ACT intervention<br>• **ACT components:**<br>○ Values clarification<br>○ Present moment awareness<br>○ Observing self/ self as context<br>○ Cognitive defusion<br>○ Committed Action	• Restraint (DEBQ)<br>• External Eating (DEBQ)<br><br>Outcome Measure(s):<br><br>• Objectively measured weight<br><br>Assessment time points:<br><br>• Baseline<br>• At each session

Hawkins et al. (2018) [16]	Study design:<br><br>Ongoing two-arm pilot RCT<br><br>Country:<br><br>USA<br><br>Eligibility:<br><br>Adults (≥21y ≤65y) with a BMI between 27 and 52kg/m^2	Sex: 73% female<br><br>Age in years[1]: 45.37 (11.31)<br><br>BMI (kg/m^2)[1]: 35.5 (5.85)<br><br>Ethnicity:<br><br>American Indian/Alaska Native: 4.7%<br><br>Asian:1.9%<br><br>Black or African American: 4.7%<br><br>Multiracial: 5.6%<br><br>Other: 4.7%<br><br>Education: 76% bachelors degree or higher<br><br>Income: n/a	Comparator (N = 54):<br><br>● **Type:** Standard behavioural treatment (SBT) + Cognitive behavioural therapy (CBT) elements<br>● **Aim:** Weight loss/ diet and physical activity<br>● **Intensity and delivery:** 23 weekly group sessions delivered over a period of 6 months<br>● **Content:** SBT based on the LEARN and Diabetes Prevention Programm weight loss and maintenance protocols, including nutrition and physical activity education, and behavioural strategies like self-monitoring, stimulus control, behavioural analysis, relapse prevention, encouraging social support + CBT including distraction and confrontation, identification of cognitive distortions, cognitive restructuring and building self-esteem (adapted from Forman et al. 2016)<br><br>Intervention (N = 54):<br><br>● **Aim:** Weight loss/ diet and physical activity<br>● **Intensity and delivery:** As above	EBT Measure(s):<br><br>● Emotional Eating (EES)<br><br>Outcome Measure(s):<br><br>● Objectively measured weight<br><br>Assessment time points:<br><br>● Baseline<br>● 6 months (end of intervention)<br>● 12 months (6-month follow-up)

			Content: SBT components similar to the above (without the CBT elements) + ACT **ACT components:** - Value clarification - Present moment awareness - Cognitive defusion (urge surfing)	
Iturbe et al. (2021) [17,18]	Study design:<br><br>Ongoing two-arm RCT<br><br>Country:<br><br>Spain<br><br>Eligibility:<br><br>Adults (≥20y ≤70y) with a BMI ≥25kg/m^2	Sex: 70.75% female<br><br>Age in years[1]: 50.65 (10.66)<br><br>BMI (kg/m^2)[1]: 37.43 (6.77)<br><br>Ethnicity: n/a<br><br>Education: n/a<br><br>Income: Average annual neighbourhood income 24590.1 € (4180.8)	Comparator (N = 78): **Type:** Usual care **Aim:** Weight loss/ diet and physical activity **Intensity and delivery:** 5 monthly one-to-one sessions of 30 mins duration, delivered by a nutritionist **Content:** Nutrition counselling, including eating and physical activity-related instructions and recommendations, seasonally adapted weekly menu, nutritional labelling, recommendations for maintaining healthy habits <br>Intervention (N = 69): **Aim:** Health behaviours according to own values (avoiding overemphasis on weight loss)	EBT Measure(s): - Emotional eating (DEBQ) - Restraint (DEBQ) - External eating (DEBQ) Outcome Measure(s): - Objectively measured weight Assessment time points: - Baseline 1 month 2 months 3 months 4 months 5 months (end of intervention) 11 months (6-month follow-up)

			• **Intensity and delivery:** 15 group sessions with 12 to 14 participants of 2hs duration (10 weekly, 5 bi-weekly), led by two psychologists with training in third-wave therapies + monthly telephone calls for a period of 6 months after the end of active treatment<br>• **Content:** Combination of ACT- and mindfulness-based intervention following a VHL approach centering primarily on valued living<br>• **ACT components:**<br>○ Value clarification<br>○ Willingness<br>○ Cognitive Defusion (Urge surfing)<br>○ Acceptance<br>○ Observing self/ self as context<br>○ Present moment awareness<br>○ Committed Action	• 17 months (12-month follow-up)<br>• 29 months (24-month follow-up)
Järvelä-Reijonen et al. (2018) [19–21]	Study design:<br><br>Three-arm RCT (intervention arms combined for meta-analyses)	Sex: 85% female<br><br>Age in years[1]: 49.5 (7.4)<br><br>BMI (kg/m^2)[1]: 31.3 (2.9)	Comparator (N = 71):<br><br>• **Type:** Waitlist<br>• **Aim:** n/a<br>• **Intensity and delivery:** n/a<br>• **Content:** n/a	EBT Measure(s):<br><br>• Restraint (TFEQ-R18)<br>• Uncontrolled Eating (TFEQ-R18)<br>• Emotional Eating (TFEQ-R18)

	<u>Country:</u><br><br>Finland<br><br><u>Eligibility:</u><br><br>Adults (≥25y ≤60y) with symptoms of psychological distress (≥3/12 on the GHQ-12) and a BMI between 27 and 35kg/m^2	<u>Ethnicity:</u> n/a<br><br><u>Education:</u> n/a<br><br><u>Income:</u> n/a	<u>Interventions</u>:<br><br>(1) Face to face (N = 70)<br><br>● **Type:** Face to face Acceptance and Commitment Therapy (ACT)<br>● **Aim:** Health behaviours according to own values (not necessarily focussed on weight loss)<br>● **Intensity and delivery:** 6 group sessions with 6 to 12 participants of 90 mins duration over a period of 8-weeks, led by a psychologist + homework and a printed workbook<br>● **Content:** Focussed on ACT skills, with some content on mindful eating, relaxation, and physical activity. No nutrition education was included<br>● **ACT components:**<br>○ Value clarification<br>○ Cognitive Defusion (Urge surfing)<br>○ Acceptance<br>○ Observing self/ self as context<br>○ Present moment awareness<br>○ Committed Action<br><br>(2) Remote (N = 78)	<u>Outcome Measure(s):</u><br><br>● Objectively measured weight<br><u>Assessment time points:</u><br><br>● 10 weeks (end of intervention)<br>● 36 weeks (6-month follow-up)

			● **Type:** Remotely delivered Acceptance and Commitment Therapy (ACT)<br>● **Aim:** Health behaviours according to own values (not necessarily focussed on weight loss)<br>● **Intensity and delivery:** One initial face to face group session to explain principles of ACT and the pre-installed Oiva mobile app. The app contained 46 exercises of approx. 1-3 mins. which participants could complete as many times they wanted in any order over an intervention period of 8-weeks<br>● **Content:** As above<br>● **ACT components:** As above	
Levin et al. (2021) [22]	Study design:<br><br>Two-arm RCT<br><br>Country:<br><br>USA<br><br>Eligibility:<br><br>Adults (≥18y) with a BMI ≥25kg/m^2	Sex: 82.3% female<br><br>Age in years[1]: 39.56 (12.12)<br><br>BMI (kg/m^2)[1]: 33.78 (5.69)<br><br>Ethnicity: 92.4% White<br><br>Education: median education: bachelors degree<br><br>Income:<br><br>Intervention median household income: $60,000-79,000	Comparator (N = 40):<br><br>● **Type:** Waitlist<br>● **Aim:** n/a<br>● **Intensity and delivery:** n/a<br>● **Content:** n/a<br><br>Intervention (N = 39):<br><br>● **Aim:** improving diet and physical activity (moving away from narrow focus on weight loss)<br>● **Intensity and delivery:** programme using text, videos and interactive	EBT Measure(s):<br><br>● Restraint (TFEQ-18)<br>● Uncontrolled Eating (TFEQ-18)<br>● Emotional Eating (TFEQ-18)<br><br>Outcome Measure(s):<br><br>● Self-reported weight<br><br>Assessment time points:

		Control median household income: $60,000-79,000	exercises delivered through an online learning management system (Canvas) monitored by a doctoral student in clinical/ counselling psychology. Included 8 weekly sessions and 5 to 10 minute weekly coaching calls or emails<br>● **Content:** Nutrition education, behavioural strategies to increase consumption of certain food groups and increase physical activity + ACT<br>● **ACT components:**<br>○ Cognitive defusion<br>○ Acceptance<br>○ Values clarification<br>○ Present moment awareness<br>○ Committed action	● Baseline<br>● 8 weeks (end of intervention)<br>● 16 weeks (2-month follow-up)
Lillis et al. (2016) [23]	Study design:<br>Two-arm RCT<br>Country:<br>USA<br>Eligibility:<br>Adults (≥18y ≤70y) with high internal disinhibition (≥5 (women) or (≥4 (men) on the TFEQ-51) and a BMI	Sex: 85% female<br>Age in years[1]: 50.2 (10.9)<br>BMI (kg/m^2)[1]: 37.6 (5.3)<br>Ethnicity:<br>Black = 5%<br>Hispanic = 6%<br>Asian = 1%<br>Caucasian = 88%<br>Education:	Comparator (N = 81):<br>● **Type:** SBT + cognitive restructuring<br>● **Aim:** Weight loss<br>● **Intensity and delivery:** approx.35 group sessions with 15 to 16 participants of 1h duration over a period of 12 months (6 months weekly, 3 months bi-weekly, 3 months monthly), run by co-leader pairs of PhD psychologists, PhD exercise physiologists and masters level nutritionists (everyone had	EBT Measure(s):<br>● Restraint (TFEQ-51)<br>● Disinhibition (TFEQ-51)<br>● Hunger (TFEQ-51)<br>Outcome Measure(s):<br>● Objectively measured weight<br>Assessment time points:<br>● Baseline<br>● 6 months (mid-intervention)

	between 30 and 50kg/m^2	Highschool = 7.5%<br><br>Some college/ vocational = 28%<br><br>Bachelors degree = 36%<br><br>Graduate or professional = 28%<br><br><u>Income:</u> n/a	training in ACT and experience with SBT)<br>● **Content:** SBT including self-monitoring, stimulus control, problem solving and goal setting + cognitive restructuring including stopping/ replacing negative thoughts, distraction techniques, relaxation skills, and environmental control methods<br><br><u>Intervention (N = 81):</u><br><br>● **Aim:** Weight loss<br>● **Intensity and delivery:** As above<br>● **Content:** SBT including self-monitoring, stimulus control, problem solving and goal setting + ACT<br>● **ACT components:**<br>○ Present moment awareness<br>○ Cognitive defusion<br>○ Observing self/ self as context<br>○ Acceptance<br>○ Values clarification<br>○ Committed action	● 12 months (end of intervention)<br>● 18 months (6-month follow-up)<br>● 24 months (12-month follow-up)

Lillis et al. (2021) [24]	Study design:<br><br>Ongoing two-arm RCT<br><br>Country:<br><br>USA<br><br>Eligibility:<br><br>Adults (≥25y ≤70y) with a BMI between 27.5 and 45kg/m^2 who lost more than 4kg in a preceding online weight loss program	Sex: 69.12% female<br><br>Age in years[1]: 57.43 (10.07)<br><br>BMI (kg/m^2)[1]: 34.36 (4.32)<br><br>Ethnicity:<br><br>Black = 2%<br><br>Hispanic = 2%<br><br>Native American = 1%<br><br>Biracial = 4%<br><br>Caucasian = 91%<br><br>Education:<br><br>Highschool = 7%<br><br>Some college/ vocational = 22%<br><br>Bachelors degree = 34%<br><br>Graduate or professional = 36%<br><br>Income: n/a	In phase 1, all participants received standard behavioural treatment (SBT). In phase 2, participants who lost more than 4kg were randomized into one of two conditions:<br><br>Comparator (N = 34):<br><br>● **Type:** Extended SBT for weight loss maintenance<br>● **Aim:** Prevent weight re-gain<br>● **Intensity and delivery:**<br>○ Phase 1: 12 weekly online lessons including 10 to 15 min interactive exercises + website for self-monitoring + weekly automated feedback<br>○ Phase 2: 4 in-person group sessions with 6 to 10 participants of 2.5 hours duration over a period of 4 months after phase 1 + 6 months of weekly emails containing reminders and micro interventions (1 to 7 mins duration) + monthly feedback emails<br>● **Content:** Extends and builds on skills taught in SBT. Participants will be taught the 3 key components of self-regulation: self-observation, self-evaluation, self-reinforcement, as	EBT Measure(s):<br><br>● Restraint (TFEQ-51)<br>● Disinhibition (TFEQ-51)<br><br>Outcome Measure(s):<br><br>● Objectively measured weight<br><br>Assessment time points:<br><br>● Phase 1 Baseline<br>● Phase 2 Baseline<br>● 6 months (end of intervention)<br>● 12 months (6-months follow-up)<br>● 18 months (12-months follow-up)<br>● 24 months (18 month-follow-up)<br>● 30 months (24-month follow-up

			well as strategies to support successful implementation of these components<br><br>Intervention (N = 34):<br>● **Aim:** prevent weight re-gain<br>● **Intensity and delivery:** As above<br>● **Content:** SBT (phase1) + ACT skills for weight loss maintenance taught in the context of how they affect momentary decisions related to food and activity.<br>● **ACT components:**<br>○ Value clarification<br>○ Acceptance<br>○ Cognitive Defusion<br>○ Present moment awareness<br>○ Committed Action	
Mueller et al. (2022) [25,26]	Study design:<br>Two-arm RCT<br><br>Country:<br>United Kingdom	Sex: 78.1% female<br><br>Age in years[1]: 50.3 (13.8)<br><br>BMI (kg/m^2)[1]: 34.8 (7.7)	Comparator (N = 196):<br>● **Type:** Waitlist, usual care<br>● **Aim:** Maintain weight loss<br>● **Content:** standard advice in the form of a leaflet from the European Association on the Study of Obesity on diet, physical activity, and mood	EBT Measure(s):<br>● Restraint (TFEQ-R21)<br>● Uncontrolled Eating (TFEQ-R21)<br>● Emotional Eating (TFEQ-R21)<br><br>Outcome Measure(s):

	Eligibility:<br><br>Adults (≥18y) with a BMI ≥ 25kg/m^2	Ethnicity:<br><br>White: 93.8%<br><br>Non-White: 5.2%<br><br>None of these: 0.3%<br><br>Prefer not to say: 0.8%<br><br>Education:<br><br>Below post-secondary (up to and including A-levels): 25.8%<br><br>Post-secondary: 72.7%<br><br>Other: 1.3%<br><br>Prefer not to say: 0.3%<br><br>Income: n/a	during the COVID 19 pandemic, tailored to people living with obesity<br>● **Intensity and delivery:** leaflet posted to participants home<br><br>Intervention (N = 192):<br><br>● **Aim:** Prevent weight gain by helping participants to manage their eating behaviours, be more physically active, and protect their emotional well-being.<br>● **Intensity and delivery:** Online self-help programme containing 12 weekly online modules + weekly automated email reminders + two semi-structured phone calls of approx. 20 minutes with trained non-specialist coaches + tailored emails after session 4 and 10<br>● **Content:** Guided self-help ACT-based weight management intervention with behavioural strategies including goal setting, self-monitoring, planning, social support, sleep hygiene, strategies to prevent lapses<br>● **ACT components:**<br>○ Acceptance<br>○ Present moment awareness<br>○ Willingness	● Self-reported weight<br><br>Assessment time points:<br><br>● 4 months (end of intervention)<br>● 12 months (8-month follow-up)

			○ Value clarification<br>○ Cognitive defusion<br>○ Comitted Action<br>● Present moment awareness	
Palmeira et al. (2017) [27,28]	Study design:<br>Two-arm RCT<br>Country:<br>Portugal<br>Eligibility:<br>Women with a BMI ≥ 25kg/m^2 enrolled in nutritional treatment for weight loss in primary care units and Hospitals from Coimbra, Portugal	Sex: 100% female<br>Age in years[1]:<br>Comparison: 42.73 (8.36)<br>Intervention: 41.97 (8.79)<br>BMI (kg/m^2)[1]:<br>Comparison: 33.65 (4.83)<br>Intervention: 34.82 (5.26)<br>Ethnicity: not reported<br>Education years[1]: 15.6 (3.21)<br>Income:	Comparator (N = 37):<br>● **Type:** Usual care, waitlist<br>● **Aim:** Weight loss/ diet and physical activity<br>● **Intensity and delivery:** individual appointments. No further details reported<br>● **Content:** Individual medical and nutritional appointments. Medical appointments include physical examination, addressing comorbidities, discussing difficulties regarding weight loss plans. Nutritional appointments include weighing, tailored dietary recommendations (according to one's needs and food preferences) and physical activity prescriptions. No psychological treatment was included.	EBT Measure(s):<br>● Restraint (TFEQ-R21)<br>● Uncontrolled Eating (TFEQ-R21)<br>● Emotional Eating (TFEQ-R21)<br>Outcome Measure(s):<br>● Objectively measures weight<br>Assessment time points:<br>● 14 weeks (end of intervention)

		84.9% low to medium socio-economic status	Intervention (N = 36):<br>● **Aim:** reduce weight self- stigma and unhealthy eating behaviors and promote quality-of-life by targeting weight-related experiential avoidance and self- criticism (without necessarily focusing on weight loss)<br>● **Intensity and delivery:** 12 (10 weekly plus 2 fortnightly) group sessions with 10-12 participants of 2 hour 30 mins duration, led by a clinical psychologist with previous training in contextual-behavioural therapies and one clinical psychology master student + a printed manual including targeted constructs, examples, and exercise sheets + audio files for mindfulness ad compassion exercises between sessions<br>● **Content:** Usual care + ACT<br>● **ACT components:**<br>○ Acceptance<br>○ Cognitive Defusion<br>○ Willingness<br>○ Present moment awareness<br>○ Values Clarification<br>○ Committed Action	

[1] mean (SD)

Abbreviations: RCT, Randomised controlled trial; BMI, Body mass index; N, Number of participants; n/a not available or not applicable

Abbreviations Questionnaires: TFEQ, Three Factor Eating Questionnaire (either 18, 21, or 51 item versions respectively); EES, Emotional Eating Scale; DEBQ, Dutch Eating Behaviour Questionnaire

7.2 Study characteristics of studies not providing IPD

Table SM 7-2: Detailed characteristics of eligible studies that were excluded and did not provide IPD

Study	Methods	Participant characteristics	Intervention and comparators	Exposures, outcomes and time points
Afari et al. (2019) [1–3]	Study design:<br><br>Two-arm RCT<br><br>Country:<br><br>USA<br><br>Eligibility:<br><br>US veterans (≥18y ≤75y) with a BMI ≥25kg/m^2 who reported "stress-related eating" (no cut-off) and attended at least 5 of 8 sessions or the equivalent of a remote preceding usual care weight loss program (MOVE! Or TeleMOVE)	Sex: 23.9% female<br><br>Age in years[1]: 57.3 (9.9)<br><br>BMI (kg/m^2)[1]: n/a<br><br>Ethnicity:<br><br>White: 70.5%<br><br>African-American: 19.3%<br><br>Hispanic: 13.6%<br><br>Education:<br><br>Highschol: 4.5%<br><br>Some college: 29.5%<br><br>Technical/ Vocational school: 23.9%<br><br>Bachelors Degree: 14.8%<br><br>Graduate/ professional: 26.1%<br><br>Income: n/a	Both groups received the MOVE! treatment before being randomised to one oft wo conditions:<br><br>Comparator (N = 43):<br><br>● **Type:** Usual care + SBT<br>● **Aim:** Weight loss<br>● **Intensity and delivery:**<br>○ MOVE!: 8 weekly group sessions or 90-day TeleMOVE programme<br>○ SBT: 4 weekly group sessions of 2h duration delivered by 3 therapists (one staff psychologist, one psychology postdoctoral fellow, and one psychology masters student)<br>● **Content:** Usual care (MOVE!) containing health education, dietary and physical activity recommendations + Standard cognitive-behavioural techniques, including goal setting, self-esteem,	EBT Measure(s):<br><br>● Emotional eating (DEBQ)<br>● External eating (DEBQ)<br>● Restraint (DEBQ)<br>Outcome Measure(s):<br><br>● Either self-reported or objectively measured weight<br>Assessment time points:<br><br>● Baseline (after MOVE!, before ransomisation)<br>● 4 weeks (end of intervention)<br>● 3-month post-intervention follow-up<br>● 6-month post-intervention follow-up

			self-monitoring, changing distorted thinking<br><br><u>Intervention (N = 45):</u><br>● **Aim:** Weight loss + disinhibited and binge eating<br>● **Intensity and delivery:** As above but stressed importance of at-home assignments and delivered by 4 therapists, one staff psychologist, two psychology postdoctoral fellows and one psychology masters student. Therapists had all received ACT training and attended weekly supervision<br>● **Content:** Usual care (MOVE!) + ACT<br>● **ACT components:**<br>○ Present moment awareness<br>○ Willingness<br>○ Values clarification<br>○ Committed Action<br>○ Observing self/ Self as context<br>○ Cognitive defusion<br>○ Acceptance	

[1] mean (SD)

Abbreviations: RCT, Randomised controlled trial; BMI, Body mass index; N, Number of participants; n/a not available or not applicable ***Abbreviations Questionnaires:*** TFEQ, Three Factor Eating Questionnaire (either 18, 21, or 51 item versions respectively); EES, Emotional Eating Scale; DEBQ, Dutch Eating Behaviour Questionnaire

8.0 Number of excluded observations and reasons

Table SM 8-1: Number of excluded observations and reasons for exclusion as indicated by original study authors

Study	Group	N	Excluded reason
Ahern 2022	Control	1	Withdrawn
	Intervention	8	Withdrawn
Butryn 2017	Control	6	No longer meeting eligibility criteria [a]
	Intervention	3	No longer meeting eligibility criteria [a]
Butryn 2022	Control	2	No longer meeting eligibility criteria [a]
	Intervention	1	No longer meeting eligibility criteria [a]
Forman 2013	Control	0	-
	Intervention	0	-
Forman 2016	Control	0	-
	Intervention	0	-
Forman 2021	Control	1	No longer meeting eligibility criteria [a]
	Intervention	2	No longer meeting eligibility criteria [a]
Frayn 2020	Control	0	-
	Intervention	0	-
Hawkins 2018	Control	0	-
	Intervention	0	-
Iturbe 2021	Control	0	-
	Intervention	1	Biologically implausible values
		1	No longer meeting eligibility criteria [a]
Järvelä-Reijonen 2018 [c]	Control	13	No longer meeting eligibility criteria [a]
		1	Unknown [b]
	Intervention	21	No longer meeting eligibility criteria [a]
Levin 2021	Control	0	-
	Intervention	0	-
Lillis 2016	Control	0	-
	Intervention	0	-
Lillis 2021	Control	0	-
	Intervention	0	-
Mueller 2022	Control	1	Biologically implausible values
	Intervention	18	Withdrawn
Palmeira 2019	Control	5	Withdrawn
	Intervention	9	Withdrawn
**Total**	Control	**6**	**Withdrawn**
		**22**	**No longer meeting eligibility criteria [a]**
		**1**	**Unknown [b]**
		**1**	**Biologically implausible values**
	Intervention	**35**	**Withdrawn**
		**28**	**No longer meeting eligibility criteria [a]**
		**1**	**Biologically implausible values**

Abbreviations: N, Number of observations

Note: All exclusions other than for the reason of biologically implausible values were performed by original trial authors and carried over to the IPD dataset.

[a] Participants were excluded by original trial authors if they would no longer have met respective eligibility criteria, for example due to pregnancy

[b] Participants were excluded by original trial authors without available details on the exclusion reason

[c] In this trial, due to the multicentre design conditions, participants were randomised before conducting baseline measurements, leading to exclusions after randomsiation

9.0 Participant Characteristics of included studies

Table SM 9-1*: Participant characteristics in included studies as derived from individual participant data*

Study	Group	N	Sex/ gender		Age in years		Baseline weight in kg	
			%female	N missing	Mean (SD)	N missing	Mean (SD)	N missing
Ahern 2022	Control	25	89%	6	46 (12.06)	6	108.05 (26.37)	6
	Intervention	33	88%	0	49 (15.55)	0	102.98 (18.39)	0
Butryn 2017	Control	175	76%	0	54 (9.45)	1	96.9 (18.07)	0
	Intervention	99	82%	0	53 (9.4)	1	96.47 (15.81)	0
Butryn 2022	Control	213	78%	0	53 (10.34)	0	96.84 (16.36)	0
	Intervention	104	78%	0	53 (10.3)	0	98.61 (18.67)	0
Forman 2013	Control	54	81%	0	45 (12.77)	0	92.03 (15.08)	0
	Intervention	74	88%	0	46 (12.91)	0	94.62 (15.32)	0
Forman 2016	Control	90	82%	0	52 (10.16)	0	101.46 (19.32)	0
	Intervention	100	82%	0	52 (9.97)	0	100.01 (18.52)	0
Forman 2021	Control	33	85%	0	51 (9.93)	0	97.55 (17.63)	0
	Intervention	240	86%	0	52 (11.07)	0	99.49 (17.24)	0
Frayn 2020	Control	43	97%	12	47 (15.18)	12	96.09 (25.66)	0
	Intervention	44	89%	9	47 (14.21)	9	96.93 (25.85)	0
Hawkins 2018	Control	54	72%	0	46 (11)	0	99.3 (20.96)	2
	Intervention	53	74%	0	45 (11.72)	0	101.25 (20.6)	0
Iturbe 2021	Control	78	67%	0	51 (10.96)	0	97.81 (19.95)	0
	Intervention	67	75%	0	50 (10.37)	0	106.03 (21.31)	0
Järvelä-Reijonen 2018	Control	71	82%	0	49 (7.4)	0	88.34 (11.5)	0
	Intervention	148	86%	0	50 (7.45)	0	87.31 (10.38)	0
Levin 2021	Control	40	82%	0	41 (14.28)	0	92.53 (15.1)	0
	Intervention	39	82%	0	38 (9.37)	0	97.1 (20.25)	0
Lillis 2016	Control	81	85%	0	50 (10.65)	0	102.2 (17.7)	0
	Intervention	81	85%	0	51 (11.25)	0	102.47 (17.27)	0
Lillis 2021	Control	34	68%	0	55 (11.11)	0	95.44 (14.88)	0

	Intervention	34	71%	0	60 (8.49)	0	97.04 (18.23)	0
Mueller 2022	Control	195	78%	1	50 (13.26)	0	97.58 (24.07)	0
	Intervention	174	79%	0	50 (14.22)	0	98.62 (21.82)	0
Palmeira 2019	Control	32	100%	0	44 (7.95)	0	88.56 (12.5)	0
	Intervention	27	100%	0	42 (9.35)	0	91.65 (16.37)	0
**Total**	Control	1218	79%	19	50 (11.49)	19	96.89 (19.21)	8
	Intervention	1317	83%	9	50 (11.65)	10	97.77 (18.64)	0

Abbreviations: N, Number of; SD, Standard deviation

Table SM 9-2 Participant weight change in included studies as derived from individual participant data

Study	Group	N	Percentage weight change from baseline to intervention end		Percentage weight change from baseline to 6 months post intervention end		Percentage weight change from baseline to 12 months post intervention end	
			Mean (SD)	N missing	Mean (SD)	N missing	Mean (SD)	N missing
Ahern 2022	Control	25	1.84 (6.83)	7	-	-	-	-
	Intervention	33	-2.53 (6.77)	4	-	-	-	-
Butryn 2017	Control	175	-10.51 (7.82)	29	-8.45 (8.21)	37	-6.73 (7.6)	40
	Intervention	99	-10.84 (7.08)	14	-8.44 (7.66)	18	-7.27 (7.47)	20
Butryn 2022	Control	213	-10.87 (10.78)	56	-8.48 (8.7)	53	-	-
	Intervention	104	-9.85 (10.73)	26	-7.57 (8.52)	31	-	-
Forman 2013	Control	54	-12.9 (6.18)	14	-11.17 (7.1)	19	-	-
	Intervention	74	-12.85 (7.75)	15	-10.93 (7.7)	25	-	-
Forman 2016	Control	90	-10.62 (8.52)	18	-	-	-5.83 (8.27)	25
	Intervention	100	-13.77 (7.94)	15	-	-	-7.96 (9.8)	22
Forman 2021	Control	33	-7.92 (7.15)	3	-	-	-2.7 (7.97)	17
	Intervention	240	-9.68 (7.72)	36	-	-	-6.35 (7.6)	117
Frayn 2020	Control	43	3.14 (28.38)	22	-	-	-	-
	Intervention	44	1.56 (8.71)	25	-	-	-	-
Hawkins 2018	Control	54	-6.33 (5.64)	11	-4.09 (6.35)	16	-	-
	Intervention	53	-8.37 (7.15)	8	-6.83 (8.07)	15	-	-

Iturbe 2021	Control	78	-4.27 (5.28)	15	-2.5 (5.1)	14	-2.48 (4.6)	27
	Intervention	67	-5.98 (5.27)	4	-4.99 (7.55)	5	-4.38 (8.96)	15
Järvelä-Reijonen 2018	Control	71	-0.2 (1.77)	3	-	-	-	-
	Intervention	148	-0.62 (2.49)	11	-	-	-	-
Levin 2021	Control	40	-0.89 (3.16)	1	-	-	-	-
	Intervention	39	-2.17 (3.57)	4	-	-	-	-
Lillis 2016	Control	81	-9.27 (8.6)	12	-6.17 (7.36)	18	-3.27 (6.48)	18
	Intervention	81	-9.06 (8.54)	14	-6.29 (8.49)	20	-5.32 (8.01)	19
Lillis 2021	Control	34	-11.09 (4.94)	2	-7.95 (5.74)	3	-5.77 (6.1)	5
	Intervention	34	-12.19 (5.28)	2	-8.58 (6.97)	2	-8.6 (8.94)	8
Mueller 2022	Control	195	-1.62 (6.87)	17	-	-	-	-
	Intervention	174	-2.01 (4.78)	29	-	-	-	-
Palmeira 2019	Control	32	-0.05 (2.4)	0	-	-	-	-
	Intervention	27	-2.1 (2.98)	0	-1.6 (3.61)	4	-	-
**Total**	Control	1218	-6.48 (9.58)	210	-7.31 (7.95)	160	-5.1 (7.25)	132
	Intervention	1317	-7.11 (8.18)	207	-7.25 (8)	120	-6.57 (8.38)	201

Abbreviations: N, Number of; SD, Standard deviation

10.0 Baseline EBTs of included studies

Table SM 10-1: Baseline eating behaviour traits in included studies as derived from individual participant data

Study	Group	N	Emotional eating		External eating/ disinhibition		Internal disinhibition		Restraint		Uncontrolled eating	
			Mean (SD)	N missing	Mean (SD)	N missing	Mean (SD)	N missing	Mean (SD)	N missing	Mean (SD)	N missing
Ahern 2022	Control	25	64 (13.65)	6	-	-	-	-	43 (18.57)	6	54 (23.52)	6
	Intervention	33	59 (18.35)	0	-	-	-	-	57 (15.91)	0	46 (23.41)	0
Butryn 2017	Control	175	56 (25.86)	1	53 (17.4)	2	53 (16.23)	1	45 (16.42)	1	42 (17.67)	1
	Intervention	99	52 (27.08)	1	51 (18.42)	2	50 (17.12)	1	46 (19.14)	1	40 (19)	1
Butryn 2022	Control	213	10 (12.39)	9	56 (24.39)	4	45 (27.86)	4	-	-	-	-
	Intervention	104	14 (14.05)	3	58 (24.85)	2	53 (31.91)	2	-	-	-	-
Forman 2013	Control	54	48 (23.76)	0	61 (24.9)	0	53 (26)	0	45 (19.75)	0	-	-
	Intervention	74	44 (21.91)	0	60 (25.24)	0	56 (26.76)	0	43 (20.98)	0	-	-
Forman 2016	Control	90	59 (27.09)	0	50 (16.94)	0	56 (16.51)	0	45 (15.78)	0	41 (17.57)	0
	Intervention	100	57 (27.4)	2	50 (15.97)	2	54 (17.64)	2	47 (16.66)	2	38 (17.02)	2
Forman 2021	Control	33	55 (24.43)	0	47 (17.3)	0	42 (11.42)	0	45 (18.18)	0	38 (19.68)	0
	Intervention	240	57 (26.23)	0	48 (16.55)	0	42 (11.51)	0	42 (15.03)	0	40 (17.21)	0
Frayn 2020	Control	43	77 (13.19)	0	64 (16.13)	0	-	-	54 (15.98)	0	-	-
	Intervention	44	74 (12.17)	0	64 (13.9)	0	-	-	54 (17.28)	0	-	-
Hawkins 2018	Control	54	30 (19.44)	5	-	-	-	-	-	-	-	-
	Intervention	53	32 (19.53)	9	-	-	-	-	-	-	-	-
Iturbe 2021	Control	78	47 (25.81)	1	54 (16.94)	1	-	-	39 (17.14)	1	-	-
	Intervention	67	59 (27.19)	0	57 (17.65)	0	-	-	42 (15.58)	0	-	-
Järvelä-Reijonen 2018	Control	71	56 (27.86)	0	-	-	-	-	46 (15.27)	0	50 (20.91)	0
	Intervention	148	64 (26.4)	0	-	-	-	-	44 (16.32)	0	49 (19.2)	0
Levin 2021	Control	40	58 (31.38)	0	-	-	-	-	39 (17.42)	0	45 (22.65)	0
	Intervention	39	58 (27.51)	0	-	-	-	-	47 (17.55)	0	49 (20.08)	0
Lillis 2016	Control	81	-	-	78 (19.27)	0	77 (19.68)	0	42 (17.56)	0	-	-

	Intervention	81	-	-	76 (20.25)	0	78 (19.32)	0	43 (17.12)	0	-	-
Lillis 2021	Control	34	-	-	56 (21.67)	0	46 (25.54)	0	42 (22.37)	0	-	-
	Intervention	34	-	-	66 (26.57)	0	62 (30.92)	0	46 (20.07)	0	-	-
Mueller 2022	Control	195	56 (19.99)	0	-	-	-	-	44 (19.49)	0	46 (20.25)	0
	Intervention	174	56 (20.19)	0	-	-	-	-	43 (18.45)	0	47 (21.14)	0
Palmeira 2019	Control	32	56 (25.69)	0	-	-	-	-	57 (18.26)	0	46 (20.72)	0
	Intervention	27	63 (22.16)	0	-	-	-	-	53 (17.46)	0	51 (16.67)	0
**Total**	Control	1218	46 (29.31)	22	57 (21.71)	7	53 (24)	5	45 (18.05)	8	45 (19.8)	7
	Intervention	1317	53 (27.38)	15	56 (21.3)	6	53 (23.21)	5	45 (17.5)	3	44 (19.43)	3

Abbreviations: N, Number of; SD, Standard deviation

11.0 Cut-off scores for EBT Strata

Table SM 11-1 Cut-off scores for 'Low', 'Medium' and 'High' Eating Behaviour Trait Strata based on Sample Tertiles

Eating Behaviour Trait	Sex/ Gender	Low	Medium	High
Emotional Eating	Females	0-48	48-67	67-100
	Males	0-33	33-59	59-100
External Eating	Females	0-50	50-67	67-100
	Males	0-48	48-65	65-100
Internal Disinhibition	Females	0-42	42-62	62-100
	Males	0-38	38-58	58-100
Restraint	Females	0-39	39-55	55-100
	Males	0-33	33-50	50-100
Uncontrolled Eating	Females	0-37	37-52	52-100
	Males	4-33	33-52	52-93

Note Only studies classified as low risk of bias for the respective eating behaviour trait for domain 6 (data discrepancies) were used as a sample to generate tertiles

12.0 Observed relationship of EBT scores and percent weight change

Figure SM 12-1 Eating Behaviour Trait scores against percentage weight change in the overall sample

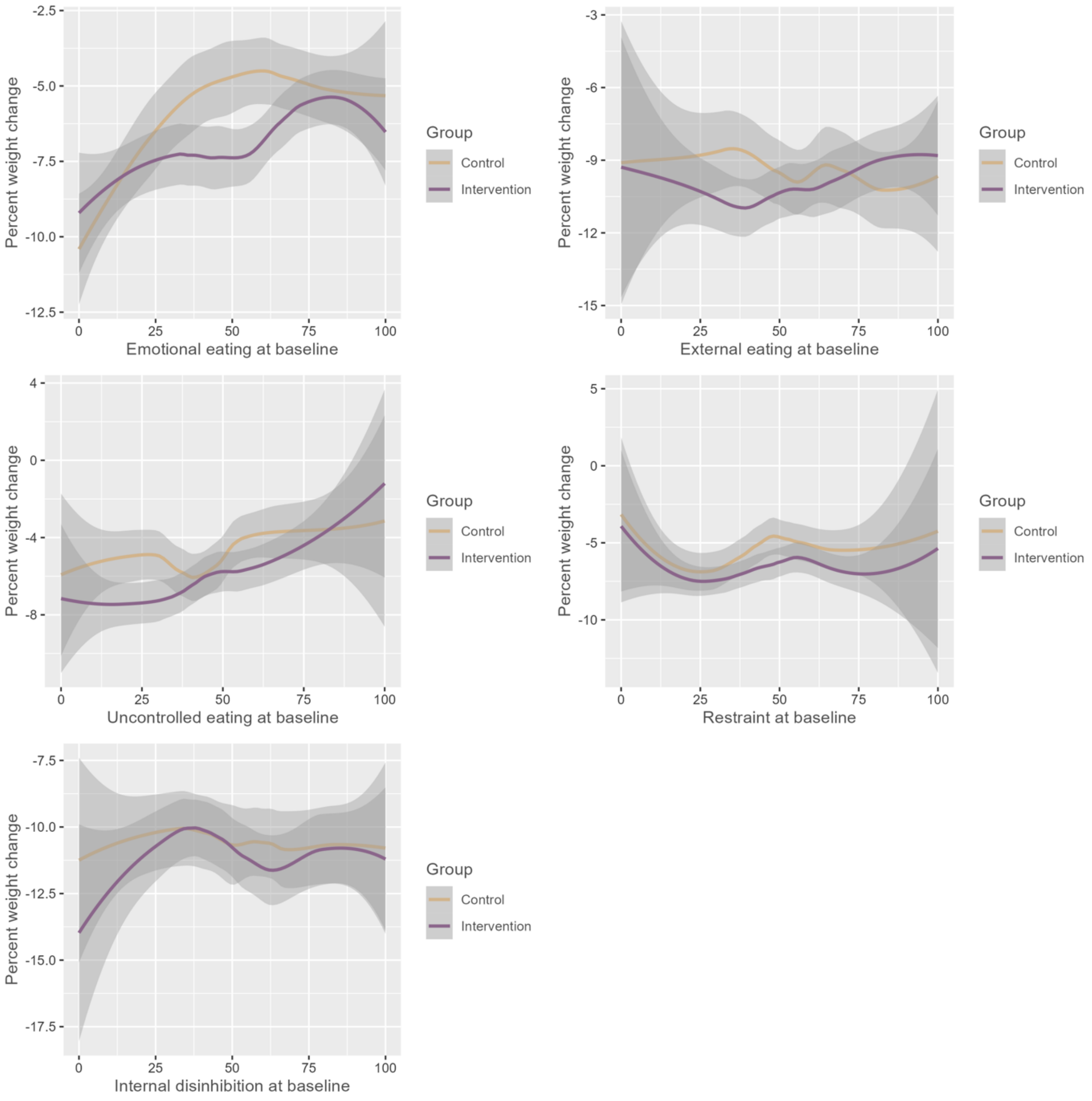

Figure SM 12-2 Eating Behaviour Trait scores against percentage weight change in trials with a standard behavioural control group

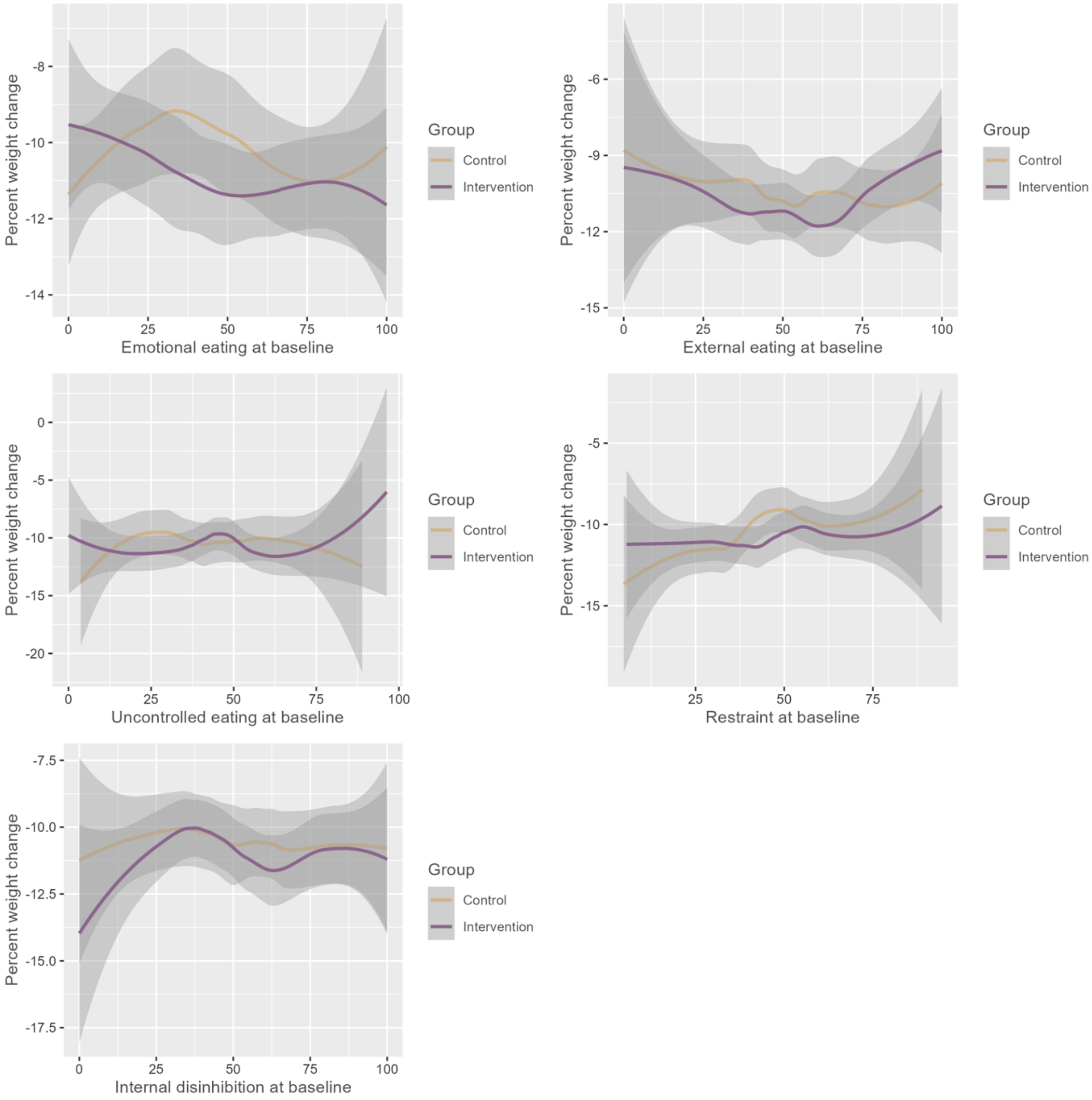

Figure SM 12-3 Eating Behaviour Trait scores against percentage weight change in trials with a minimal control group

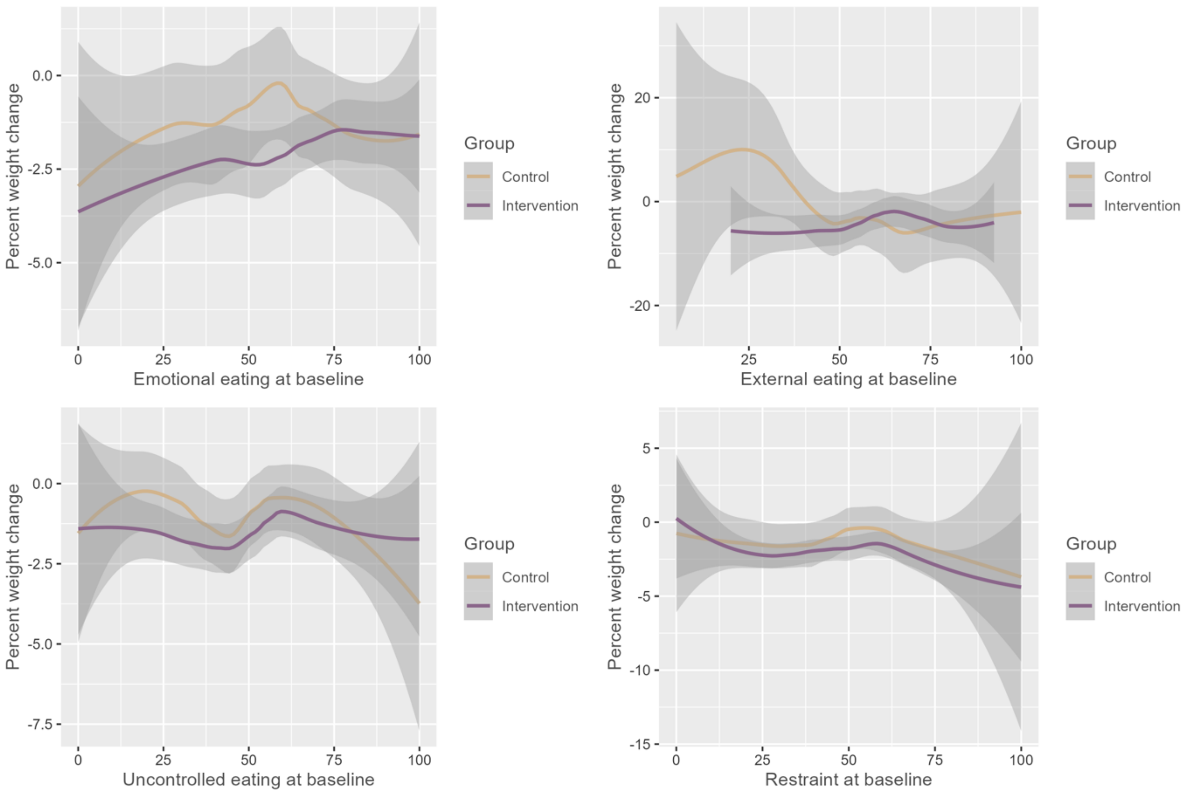

Figure SM 12-4 Eating Behaviour Trait scores against percentage weight change in trials that significantly reduced experiential avoidance

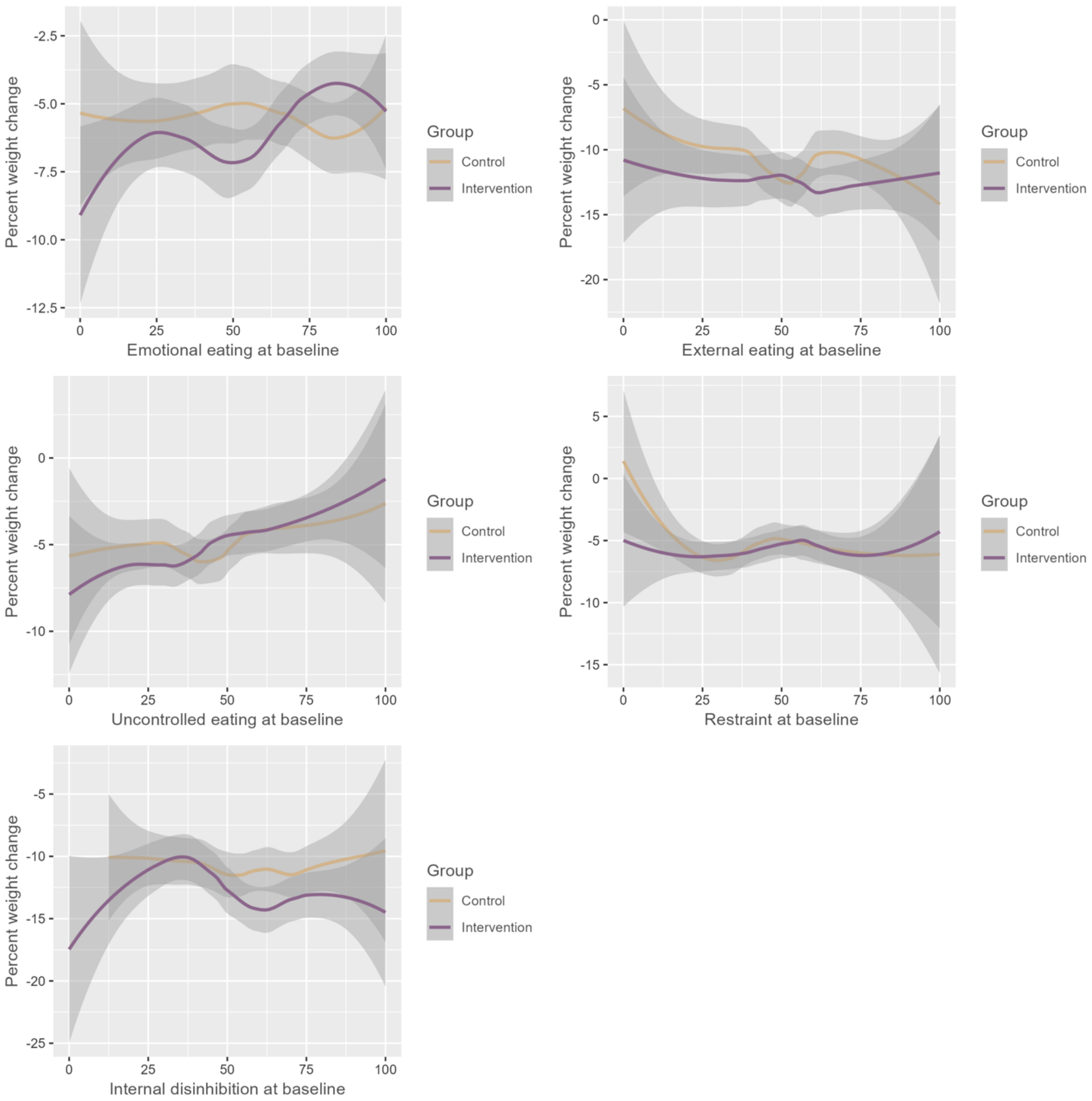

Figure SM 12-5 Eating Behaviour Trait scores against percentage weight change in trials that used the Three Factor Eating Questionnaire

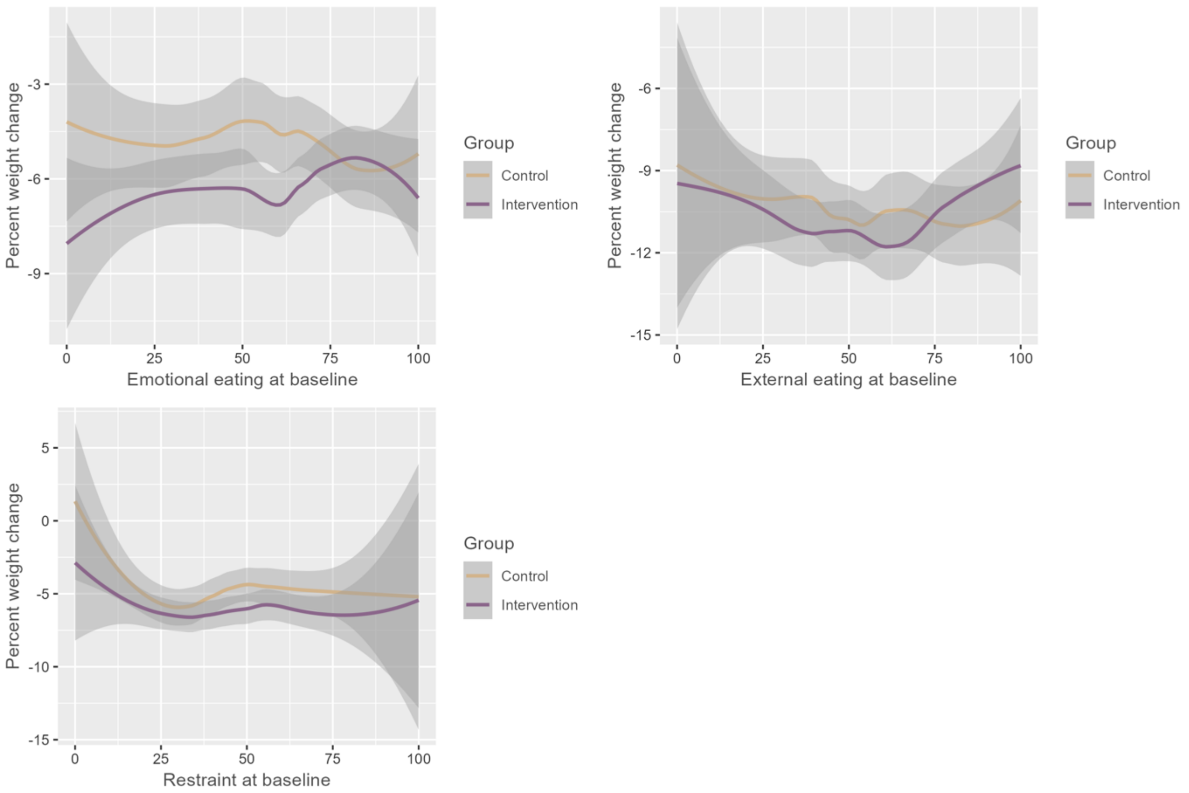

Figure SM 12-6 Eating Behaviour Trait scores against percentage weight change in participants that attended at least 60% of intervention sessions

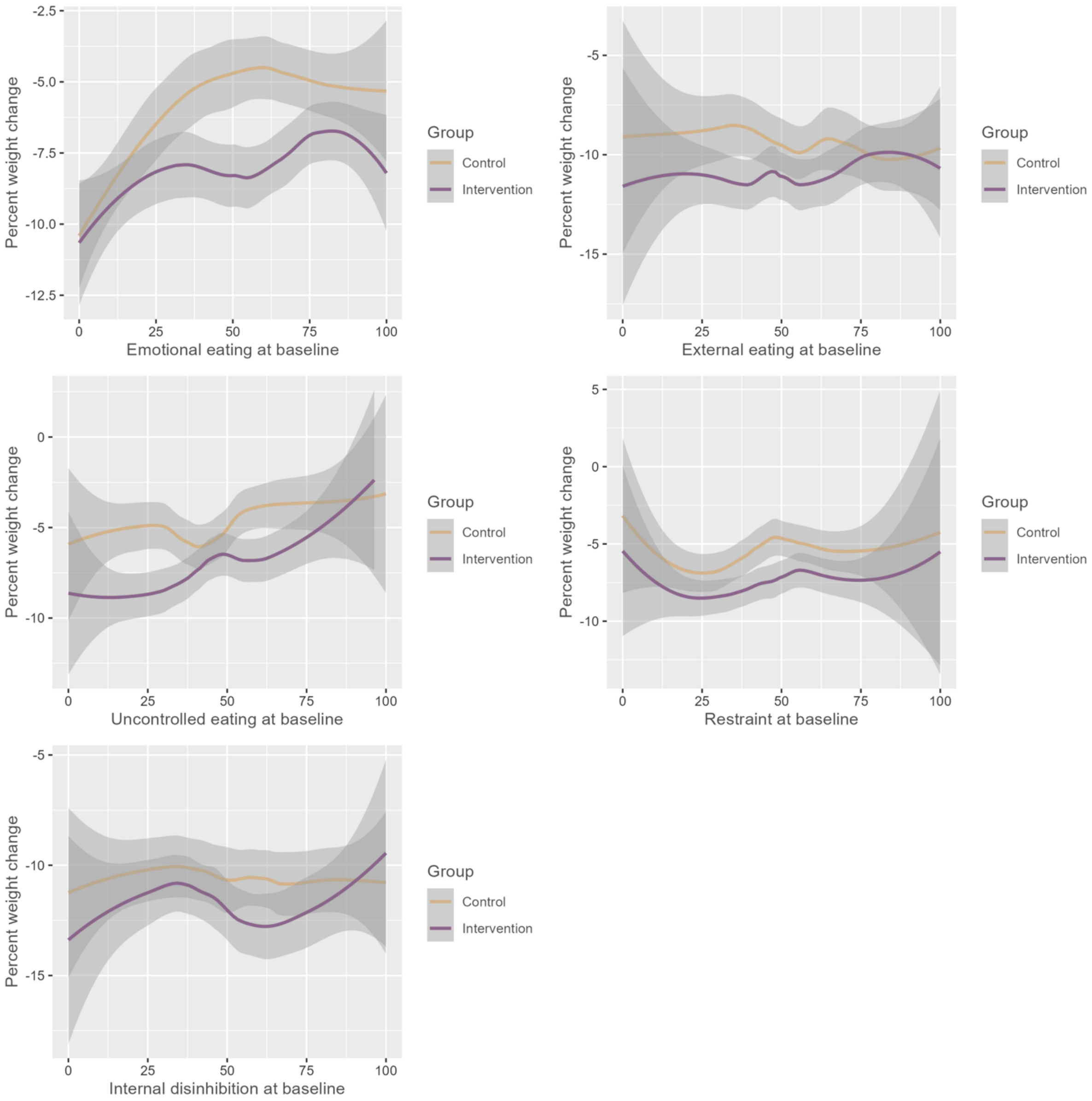

13.0 Intervention effects on weight change

Table SM 13-1 The effect of intervention on percentage weight change at end of intervention, 6 – and 12 month follow-up

Timepoint	N sample [N studies]	Difference in mean percentage weight change (95% CI) [a]	Tau^2
Intervention End	2111 [15]	-0.715 (-1.29, -0.03)	0.42
6-Months	924 [7]	-0.289 (-1.32, 0.74)	0
12-Months	778 [6]	-1.702 (-2.87, -0.53)	0

[a] *Intervention effects (ACT vs control) were estimated while stratifying all other parameters by trial*

Abbreviations: CI, Confidence Interval

Note: Rank deficient coefficients were removed from the model by the lmer function (e.g. if one study only included females and no males)

14.0 Intervention effects on changes in eating behaviour traits

Table SM 14-1 The effect of intervention on changes in eating behaviour traits from baseline to end of intervention

Eating Behaviour Trait	N sample [N studies]	Difference in mean EBT change (95% CI) [a]	Tau^2
Emotional Eating	1582 [12]	-3.629 (-5.04, -1.71)	2.45
External Eating	1016 [8]	-2.415 (-4.95, 0.12)	0.00
Internal Disinhibition	866 [6]	-3.243 (-5.93, -0.57)	0.00
Restraint	1509 [12]	2.867 (1.08, 4.71)	6.06
Uncontrolled Eating	1059 [7]	-5.753 (-9.25, -2.51)	16.69

[a] *Intervention effects (ACT vs control) were estimated while stratifying all other parameters by trial*

Abbreviations: CI, Confidence Interval

Note: Rank deficient coefficients were removed from the model by the lmer function (e.g. if one study only included females and no males

15.0 Sensitivity analyses

Table SM 15-1 Sensitivity analyses of intervention and interaction effects for emotional eating at end of intervention

Sensitivity analyses	N sample [N studies]	Intervention effect on percentage weight change		Interaction of categorised EBTs and randomised group on percentage weight change		Interaction of continuous EBTs and randomised group on percentage weight change	
		MD (95% CI) [a]	Tau^2	MD (95% CI) [b, c]	Tau^2	MD (95% CI) [b]	Tau^2
Low risk of bias	828 [6]					-0.001 (-0.04, 0.03)	0.00
Low	333	-0.759 (-2.16, 0.64)	0.00	0.838 (-1.30, 2.98)	0.00		
Medium	268	-1.795 (-3.54, -0.13)	2.64	ref			
High	227	-0.466 (-2.05, 1.11)	0.00	0.559 (-1.79, 2.91)	0.00		
Minimal comparison	866 [7]					0.020 (-0.01, 0.05)	0.00
Low	249	**-4.687 (-3.47, -0.56)**	50.84	-0.534 (-2.16, 1.14)	0.07		
Medium	304	**-1.293 (-2.39, -0.16)**	0.00	ref			
High	313	-0.253 (-2.13, 1.62)	4.55	0.337 (-0.94, 3.08)	5.77		
Standard behavioural comparison	1027 [6]					-0.006 (-0.06, 0.04)	0.00
Low	560	-0.145 (-1.71, 1.42)	0.00	1.214 (-1.81, 4.24)	0.00		
Medium	261	**-2.488 (-4.7, -0.29)**	3.45	ref			
High	206	-0.187 (-2.54, 2.15)	0.00	1.200 (-2.33, 4.72)	0.00		
Reduced experiential avoidance	1134 [7]					0.015 (-0.01, 0.05)	0.00
Low	387	-0.771 (-1.99, 0.44)	0.00	0.754 (-0.97, 2.61)	0.00		
Medium	396	**-1.718 (-3.06, -0.59)**	1.85	ref			
High	351	0.123 (-0.83, 1.94)	3.15	**1.581 (0.11, 3.81)**	2.39		
TFEQ only	1328 [8]					0.019 (-0.01, 0.05)	0.00
Low	425	**-1.32 (-2.56, -0.08)**	0.00	0.653 (-1.07, 2.43)	0.00		
Medium	478	**-2.10 (-3.30, -0.91)**	1.53	ref			
High	425	-0.102 (-1.19, 1.64)	2.30	**2.069 (0.44, 4.01)**	1.27		
Sufficient dose of intervention (≥ 60%)	1621 [11]					0.008 (-0.02, 0.04)	0.00
Low	691	-0.859 (-2.12, 0.40)	0.00	0.571 (-1.32, 2.47)	0.00		

Medium	491	**-2.345 (-3.6, -1.16)**	0.98	ref			
High	439	-0.34 (-1.51, 1.02)	0.65	1.844 (-0.13, 3.81)	0.00		

[a] *Intervention effects (ACT vs control) on percentage weight change were estimated while stratifying all other parameters by trial*

[b] *Interactions between randomised group (ACT vs control) and emotional eating on percentage weight change were estimated while stratifying all other parameters by trial*

[c] *Reference group was medium levels of emotional eating*

Abbreviations: N, Number of; MD, Mean Difference; CI, Confidence Interval; TFEQ, Three Factor Eating Questionnaire

Note: Rank deficient coefficients were removed from the model by the lmer function (e.g. if one study only included females and no males)

Table SM 15-2 Sensitivity analyses of intervention and interaction effects for external eating at end of intervention

Sensitivity analyses	N sample [N studies]	Intervention effect on percentage weight change		Interaction of categorised EBTs and randomised group on percentage weight change		Interaction of continuous EBTs and randomised group on percentage weight change	
		MD (95% CI) [a]	Tau^2	MD (95% CI) [b, c]	Tau^2	MD (95% CI) [b]	Tau^2
Low risk of bias	852 [5]					0.046 (-0.01, 0.1)	0.00
Low	354	-1.193 (-3.18, 0.8)	0	-0.79 (-3.81, 2.08)	3.45		
Medium	236	-0.675 (-2.94, 1.46)	2.97	ref			
High	262	1.428 (-0.68, 3.66)	1.41	1.456 (-1.7, 4.89)	0.00		
Standard behavioural comparison	1150 [7]					0.043 (-0.01, 0.09)	0.00
Low	534	-1.296 (-2.93, 0.34)	0.00	-0.448 (-3.01, 2.02)	1.69		
Medium	309	-1.185 (-3.19, 0.73)	1.66	ref			
High	307	1.386 (-0.43, 3.42)	1.29	1.857 (-0.98, 4.86)	0.00		
TFEQ only	1150 [7]					0.043 (-0.01, 0.09)	0.00
Low	534	-1.296 (-2.93, 0.34)	0.00	-0.58 (-3.1, 1.81)	1.93		
Medium	336	-1.029 (-2.94, 0.81)	2.62	ref			
High	280	1.541 (-0.47, 3.55)	0.00	1.685 (-1.13, 4.69)	0.00		
Sufficient dose of intervention (≥ 60%)	1028 [6]					**0.050 (-0.002, 0.10)**	0.00
Low	496	**-1.906 (-3.59, -0.23)**	0.00	-0.089 (-2.7, 2.24)	3.7		
Medium	312	-1.661 (-3.54, 0.07)	3.39	ref			
High	220	0.53 (-1.67, 3.06)	4.43	2.052 (-0.77, 5.28)	0.00		

[a] *Intervention effects (ACT vs control) on percentage weight change were estimated while stratifying all other parameters by trial*

[b] *Interactions between randomised group (ACT vs control) and external eating/ disinhibition on percentage weight change were estimated while stratifying all other parameters by trial*

[c] *Reference group was medium levels of external eating/ disinhibition*

Abbreviations: N, Number of; MD, Mean Difference; CI, Confidence Interval; TFEQ, Three Factor Eating Questionnaire

Note: Rank deficient coefficients were removed from the model by the lmer function (e.g. if one study only included females and no males)

Table SM 15-3 Sensitivity analyses of intervention and interaction effects for internal disinhibition at end of intervention

Sensitivity analyses	N sample [N studies]	Intervention effect on percentage weight change		Interaction of categorised EBTs and randomised group on percentage weight change		Interaction of continuous EBTs and randomised group on percentage weight change	
		MD (95% CI) [a]	Tau^2	MD (95% CI) [b, c]	Tau^2	MD (95% CI) [b]	Tau^2
Low risk of bias	854 [5]					0.004 (-0.05, 0.06)	0.00
Low	258	-0.272 (-2.53, 2)	0.45	1.615 (-1.84, 4.49)	5.24		
Medium	284	-2.04 (-5.04, 0.93)	6.69	ref			
High	312	0.492 (-1.45, 2.58)	3.65	2.118 (-1.03, 5.08)	4.45		
Standard behavioural comparison	1152 [7]					0.008 (-0.04, 0.06)	0.00
Low	413	-0.777 (-2.56, 1.13)	0.72	0.648 (-2.07, 3.21)	1.94		
Medium	397	-1.459 (-3.29, 0.41)	4.42	ref			
High	342	0.506 (-1.23, 2.46)	3.1	1.802 (-1.04, 4.46)	1.31		
Sufficient dose of intervention (≥ 60%)	907 [5]					0.016 (-0.04, 0.07)	0.00
Low	363	-1.158 (-3.06, 0.86)	0.81	2.077 (-0.98, 4.78)	3.89		
Medium	328	**-3.125 (-5.11, -0.97)**	4.54	ref			
High	216	0.348 (-2.1, 2.98)	5.48	**3.755 (0.39, 6.7)**	4.11		

[a] *Intervention effects (ACT vs control) on percentage weight change were estimated while stratifying all other parameters by trial*

[b] *Interactions between randomised group (ACT vs control) and internal disinhibition on percentage weight change were estimated while stratifying all other parameters by trial*

[c] *Reference group was medium levels of internal disinhibition*

Abbreviations: N, Number of; MD, Mean Difference; CI, Confidence Interval; TFEQ, Three Factor Eating Questionnaire

Note: Rank deficient coefficients were removed from the model by the lmer function (e.g. if one study only included females and no males)

Table SM 15-4 Sensitivity analyses of intervention and interaction effects for restraint at end of intervention

Sensitivity analyses	N sample [N studies]	Intervention effect on percentage weight change		Interaction of categorised EBTs and randomised group on percentage weight change		Interaction of continuous EBTs and randomised group on percentage weight change	
		MD (95% CI) [a]	Tau^2	MD (95% CI) [b, c]	Tau^2	MD (95% CI) [b]	Tau^2
Low risk of bias	886 [6]					0.003 (-0.05, 0.06)	0.00
Low	355	-0.804 (-2.25, 0.69)	0.62	1.353 (-0.94, 3.64)	0.00		
Medium	229	**-2.291 (-4.07, -0.51)**	0.00	ref			
High	302	-0.266 (-1.73, 1.2)	0.00	2.118 (-1.03, 5.08)	0.00		
Minimal comparison	866 [7]					-0.004 (-0.04, 0.04)	0.00
Low	341	**-1.034 (-2.07, -0.003)**	0.00	-0.568 (-2.20, 1.07)	0.00		
Medium	244	-0.384 (-1.83, 1.06)	0.00	ref			
High	281	-1.483 (-2.07, 0.30)	5.71	-0.347 (-2.07, 1.37)	0.00		
Standard behavioural comparison	920 [6]					-0.014 (-0.07, 0.05)	0.00
Low	404	-0.758 (-2.43, 0.92)	0.08	1.617 (-1.1, 4.33)	0.00		
Medium	228	**-2.659 (-4.86, -0.45)**	0.00	ref			
High	288	-0.981 (-2.67, 0.88)	1.42	1.234 (-1.6, 4.07)	0.00		
Reduced experential avoidance	1134 [7]					0.014 (-0.03, 0.06)	0.00
Low	467	**-1.226 (-2.37, -0.08)**	0.00	0.038 (-1.82, 1.9)	0.00		
Medium	287	**-1.512 (-3.02, -0.003)**	0.00	ref			
High	380	-0.36 (-1.58, 0.86)	0.00	0.629 (-1.3, 2.56)	0.00		
TFEQ only	1328 [8]					0.000 (-0.04, 0.04)	0.00
Low	552	-1.019 (-2.19, 0.15)	0.00	0.550 (-1.13, 2.23)	0.00		
Medium	448	**-1.797 (-3.05, -0.54)**	0.00	ref			
High	328	-0.668 (-1.98, 0.74)	0.21	0.881 (-1.01, 2.77)	0.00		
Sufficient dose of intervention (≥ 60%)	1397 [10]					0.004 (-0.04, 0.04)	0.00
Low	573	**-1.639 (-2.78, -0.50)**	0.00	-0.06 (-1.8, 1.68)	0.00		
Medium	378	**-1.933 (-3.33, -0.53)**	0.00	ref			
High	446	**-1.56 (-2.53, -0.21)**	1.60	0.219 (-1.6, 2.04)	0.00		

[a] *Intervention effects (ACT vs control) on percentage weight change were estimated while stratifying all other parameters by trial*

[b] *Interactions between randomised group (ACT vs control) and restraint on percentage weight change were estimated while stratifying all other parameters by trial*

[c] *Reference group was medium levels of restraint*

Abbreviations: N, Number of; MD, Mean Difference; CI, Confidence Interval; TFEQ, Three Factor Eating Questionnaire

Note: Rank deficient coefficients were removed from the model by the lmer function (e.g. if one study only included females and no males)

Table SM 15-5 Sensitivity analyses of intervention and interaction effects for uncontrolled eating at end of intervention

Sensitivity analyses	N sample [N studies]	Intervention effect on percentage weight change		Interaction of categorised EBTs and randomised group on percentage weight change		Interaction of continuous EBTs and randomised group on percentage weight change	
		MD (95% CI) [a]	Tau^2	MD (95% CI) [b, c]	Tau^2	MD (95% CI) [b]	Tau^2
Low risk of bias	592 [4]					-0.016 (-0.07, 0.04)	0.00
Low	246	-1.344 (-3.16, 0.47)	0.00	-0.865 (-3.25, 1.52)	0.00		
Medium	200	-0.145 (-1.98, 1.69)	0.00	ref			
High	205	**-2.177 (-3.55, -0.63)**	0.80	-1.647 (-4.18, 0.88)	0.00		
Minimal comparison	707 [5]					0.022 (-0.01, 0.06)	0.00
Low	234	**-2.059 (-2.81, -0.54)**	1.89	-0.285 (-2.10, 1.53)	0.00		
Medium	191	-0.841 (-2.32, 0.64)	0.00	ref			
High	282	-0.669 (-1.47, 0.89)	1.47	0.907 (-0.83, 2.64)	0.00		
Standard behavioural comparison	601 [3]					-0.03 (-0.11, 0.05)	0.00
Low	288	-1.338 (-3.43, 0.76)	0.00	-1.137 (-4.36, 2.08)	0.00		
Medium	195	-0.208 (-2.59, 2.23)	0.46	ref			
High	138	**-3.419 (-6.18, -0.65)**	0.00	-2.955 (-6.86, 0.94)	0.00		
Reduced experiential avoidance	1035 [6]					0.009 (-0.03, 0.05)	0.00
Low	400	**-1.402 (-2.62, -0.18)**	0.00	-0.575 (-2.45, 1.3)	0.00		
Medium	293	-0.444 (-1.98, 1.09)	0.00	ref			
High	342	-1.358 (-2.83, 0.38)	2.47	-0.195 (-2.11, 1.82)	0.79		
Sufficient dose of intervention (≥ 60%)	1180 [8]					-0.002 (-0.04, 0.04)	0.00
Low	463	**-1.979 (-3.26, -0.7)**	0.00	-0.614 (-2.52, 1.29)	0.00		
Medium	348	-0.907 (-2.44, 0.62)	0.00	ref			
High	369	**-2.21 (-3.37, -0.62)**	2.32	-0.593 (-2.59, 1.41)	0.00		

[a] *Intervention effects (ACT vs control) on percentage weight change were estimated while stratifying all other parameters by trial*

[b] *Interactions between randomised group (ACT vs control) and uncontrolled eating on percentage weight change were estimated while stratifying all other parameters by trial*

[c] *Reference group was medium levels of uncontrolled eating*

Abbreviations: N, Number of; MD, Mean Difference; CI, Confidence Interval; TFEQ, Three Factor Eating Questionnaire

Note: Rank deficient coefficients were removed from the model by the lmer function (e.g. if one study only included females and no males)

16.0 References Supplementary Material

1 Afari N, Herbert MS, Godfrey KM, *et al.* Acceptance and commitment therapy as an adjunct to the *MOVE!* programme: a randomized controlled trial. *Obes Sci Pract*. 2019;5:397–407. doi: 10.1002/osp4.356

2 Wooldridge JS, Blanco BH, Dochat C, *et al.* Relationships Between Dietary Intake and Weight-Related Experiential Avoidance Following Behavioral Weight-Loss Treatment. *Int J Behav Med*. 2022;29:104–9. doi: 10.1007/s12529-021-09990-0

3 Wooldridge JS, Herbert MS, Hernandez J, *et al.* Improvement in 6-min Walk Test Distance Following Treatment for Behavioral Weight Loss and Disinhibited Eating: an Exploratory Secondary Analysis. *Int J Behav Med*. 2019;26:443–8. doi: https://dx.doi.org/10.1007/s12529-019-09796-1

4 Ahern AL, Richards R, Jones RA, *et al.* Acceptability and feasibility of an acceptance and commitment therapy-based guided self-help intervention for weight loss maintenance in adults who have previously completed a behavioural weight loss programme: The SWiM feasibility study protocol. *BMJ Open*. 2022;12. doi: 10.1136/bmjopen-2021-058103

5 Butryn ML, Forman EM, Lowe MR, *et al.* Efficacy of environmental and acceptance-based enhancements to behavioral weight loss treatment: The ENACT trial. *Obesity*. 2017;25:866–72. doi: 10.1002/oby.21813

6 Butryn ML, Crane NT, Lufburrow E, *et al.* The Role of Physical Activity in Long-term Weight Loss: 36-month Results From a Randomized Controlled Trial. *Ann Behav Med*. 2023;57:146–54. doi: 10.1093/abm/kaac028

7 Butryn ML, Godfrey KM, Call CC, *et al.* Promotion of physical activity during weight loss maintenance: A randomized controlled trial. *Health Psychology*. 2021;40:178–87. doi: 10.1037/hea0001043

8 Call CC, D'Adamo L, Crane NT, *et al.* The relation of grit to weight loss maintenance outcomes. *J Contextual Behav Sci*. 2022;24:60–4. doi: 10.1016/j.jcbs.2022.03.008

9 Forman EM, Butryn ML, Juarascio AS, *et al.* The mind your health project: A randomized controlled trial of an innovative behavioral treatment for obesity. *Obesity*. 2013;21:1119–26. doi: 10.1002/oby.20169

10 Forman EM, Manasse SM, Butryn ML, *et al.* Long-Term Follow-up of the Mind Your Health Project: Acceptance-Based versus Standard Behavioral Treatment for Obesity. *Obesity*. 2019;27:565–71. doi: 10.1002/oby.22412

11 Forman EM, Butryn ML, Manasse SM, *et al.* Acceptance-based versus standard behavioral treatment for obesity: Results from the mind your health randomized controlled trial. *Obesity*. 2016;24:2050–6. doi: 10.1002/oby.21601

12 Coffman DL, Oliva IB, Forman EM. Does acceptance-based treatment moderate the effect of stress on dietary lapses?. *Transl Behav Med*. 2021;11:2110–5. doi: https://dx.doi.org/10.1093/tbm/ibab078

13 Godfrey KM, Schumacher LM, Butryn ML, *et al.* Physical Activity Intentions and Behavior Mediate Treatment Response in an Acceptance-Based Weight Loss Intervention. *Annals of Behavioral Medicine*. 2019;53:1009–19. doi: 10.1093/abm/kaz011

14 Forman EM, Chwyl C, Berry MP, *et al.* Evaluating the efficacy of mindfulness and acceptance-based treatment components for weight loss: Protocol for a multiphase optimization strategy trial. *Contemp Clin Trials*. 2021;110. doi: 10.1016/j.cct.2021.106573

15 Frayn M, Carrière K, Knäuper B. Lessons Learned from an ACT-Based Physician-Delivered Weight Loss Intervention: A Pilot RCT Demonstrates Limits to Feasibility. *Open Psychol*. 2020;2:22–39. doi: 10.1515/psych-2020-0003

16 Hawkins MAW, Colaizzi J, Gunstad J, *et al.* Cognitive and Self-regulatory Mechanisms of Obesity Study (COSMOS): Study protocol for a randomized controlled weight loss trial examining change in biomarkers, cognition, and self-regulation across two behavioral treatments. *Contemp Clin Trials*. 2018;66:20–7. doi: 10.1016/j.cct.2017.12.010

17 Iturbe I, Pereda-Pereda E, Echeburúa E, *et al.* The effectiveness of an acceptance and commitment therapy and mindfulness group intervention for enhancing the psychological and physical well-being of adults with overweight or obesity seeking treatment: The mind&life randomized control trial study protocol. *Int J Environ Res Public Health*. 2021;18. doi: 10.3390/ijerph18094396

18 Iturbe I, Urkia-Susin I, Echeburúa E, *et al.* An acceptance and commitment therapy and mindfulness group intervention for the psychological and physical well-being of adults with body mass indexes in the overweight or obese range: The Mind&Life randomized controlled trial. *J Contextual Behav Sci*. 2024;34:100827. doi: 10.1016/j.jcbs.2024.100827

19 Sairanen E, Tolvanen A, Karhunen L, *et al.* Psychological flexibility mediates change in intuitive eating regulation in acceptance and commitment therapy interventions. *Public Health Nutr*. 2017;20:1681–91. doi: 10.1017/S1368980017000441

20 Järvelä-Reijonen E, Karhunen L, Sairanen E, *et al.* The effects of acceptance and commitment therapy on eating behavior and diet delivered through face-to-face contact and a mobile app: A randomized controlled trial. *International Journal of Behavioral Nutrition and Physical Activity*. 2018;15. doi: 10.1186/s12966-018-0654-8

21 Lappalainen R, Sairanen E, Järvelä E, *et al.* The effectiveness and applicability of different lifestyle interventions for enhancing wellbeing: The study design for a randomized controlled trial for persons with metabolic syndrome risk factors and psychological distress. *BMC Public Health*. 2014;14. doi: 10.1186/1471-2458-14-310

22 Levin ME, Petersen JM, Durward C, *et al.* A randomized controlled trial of online acceptance and commitment therapy to improve diet and physical activity among adults who are overweight/obese. *Transl Behav Med*. 2021;11:1216–25. doi: 10.1093/tbm/ibaa123

23 Lillis J, Niemeier HM, Thomas JG, *et al.* A randomized trial of an acceptance-based behavioral intervention for weight loss in people with high internal disinhibition. *Obesity*. 2016;24:2509–14. doi: 10.1002/oby.21680

24 Lillis J, Schumacher L, Thomas JG, *et al.* Study protocol for a randomized controlled trial comparing two low-intensity weight loss maintenance interventions based on acceptance

and commitment therapy or self-regulation. *Contemp Clin Trials*. 2021;103. doi: 10.1016/j.cct.2021.106327

25 Mueller J, Richards R, Jones RA, *et al.* Supporting Weight Management during COVID-19: A Randomized Controlled Trial of a Web-Based, ACT-Based, Guided Self-Help Intervention. *Obes Facts*. 2022;15:550–9. doi: 10.1159/000524031

26 Richards R, Jones RA, Whittle F, *et al.* Development of a Web-Based, Guided Self-help, Acceptance and Commitment Therapy–Based Intervention for Weight Loss Maintenance: Evidence-, Theory-, and Person-Based Approach. *JMIR Form Res*. 2022;6. doi: 10.2196/31801

27 Palmeira L, Pinto-Gouveia J, Cunha M. Exploring the efficacy of an acceptance, mindfulness & compassionate-based group intervention for women struggling with their weight (Kg-Free): A randomized controlled trial. *Appetite*. 2017;112:107–16. doi: 10.1016/j.appet.2017.01.027

28 Palmeira L, Cunha M, Pinto-Gouveia J. Processes of change in quality of life, weight self-stigma, body mass index and emotional eating after an acceptance-, mindfulness- and compassion-based group intervention (Kg-Free) for women with overweight and obesity. *J Health Psychol*. 2019;24:1056–69. doi: 10.1177/1359105316686668
